# Factors related to sickness absenteeism among Brazilian Nursing professionals before, during, and after the pandemic*

**DOI:** 10.1590/1518-8345.7696.4623

**Published:** 2025-07-28

**Authors:** Fernanda Seidel Pinheiro, Miguel Lucas Silva da Paixão, Gabriel Fernandes Gonçalves, João Lucas Campos de Oliveira, Daiane Dal Pai, Juliana Petri Tavares

**Affiliations:** 1 Universidade Federal do Rio Grande do Sul, Escola de Enfermagem, Porto Alegre, RS, Brazil.

**Keywords:** Absenteeism, Occupational Health, Nursing, COVID-19, Pandemics, Personnel Management

## Abstract

to identify factors related to sick leave absenteeism among Brazilian nursing professionals before, during, and after the COVID-19 pandemic.

a cross-sectional study involving nursing professionals from medical, surgical, intensive care, and adult emergency units, with absences recorded between 2019 and 2022. Sociodemographic, occupational, and absence-related variables were evaluated. Descriptive statistical analysis, absenteeism rate calculation, and Poisson Regression with robust variance were performed, considering p≤0.05.

a sample of 839 professionals, with 7,375 absences, was analyzed. Sick leave absenteeism resulted in an average of 54.1±2.5 lost days (p<0.001) and was more prevalent among professionals aged 41 years or younger (31.8%; p=0.003). The intensive care (31.3%) and medical inpatient (27.5%) units reported the highest number of absences. The highest absenteeism rate (9.9%) occurred in July 2020. The risk of illness was associated with male gender (p≤0.001) and intensive care unit work (p=0.007) in the 1^st^period; being single (p=0.002) and being a nursing technician (p=0.022) in the 2^nd^period; and working in intensive care (p=0.003) and as a nursing technician (p≤0.001) in the 3^rd^ period.

after the end of the pandemic, absenteeism rates did not return to pre-pandemic levels. COVID-19 and musculoskeletal diseases were the most prevalent causes. It was possible to investigate the factors related to absenteeism.

## Highlights

**(1)** The number of days absent and age ≤ 41 are associated with absenteeism. **(2)** The main causes of sick leave absenteeism were COVID-19 and musculoskeletal diseases. **(3)** Medical inpatient care had the highest absenteeism rate across all sectors and periods. **(4)** Absence during the 2nd and 3rd periods exceeded that of the 1st, but was high in all periods.

## Introduction

Work-related illness is an old but persistent issue, particularly concerning nursing professionals. Absenteeism among nursing staff is a matter of significant public health relevance, directly affecting the quality of care and the sustainability of healthcare services. It is known that sick leave absenteeism includes all absences due to illness or medical procedures, excluding occupational diseases^(1)^. When absenteeism occurs among nursing workers, it disrupts services, causes dissatisfaction and overload within the team, and leads to a decline in the quality of patient care^(2)^.

The psychosocial and psychosomatic impacts arising from the nature of nursing work reduce productivity and tend to increase trauma, emotional exhaustion, fear of contamination, and feelings such as sadness, irritability, and the desire to give up everything, contributing to increased absenteeism among these professionals in healthcare services^(3-6)^.

Among the leading causes of absenteeism in nursing are respiratory diseases, infectious and parasitic diseases, and musculoskeletal and connective tissue disorders, often resulting from occupational exposures^(7)^. Notably, mental and behavioral disorders, clinical conditions, and musculoskeletal and connective tissue diseases usually present the highest percentages^(5,8)^.

With the onset of the COVID-19 pandemic, issues affecting nursing professionals daily were exacerbated, including workload overload, long and exhausting shifts, poor sleep quality, double work shifts, inefficient work processes, and insufficient material resources, among others^(9-10)^. In response to the rapid spread of COVID-19, national and international studies reported growing concerns, including anticipation of the disease’s impact, fear of reduced or lacking Personal Protective Equipment (PPE), and worries about personal safety and the well-being of loved ones, which were alarming^(11-12)^.

Internationally, some studies identified an increased risk of severe physical morbidity and extended leave periods for nursing professionals who treated COVID-19 patients^(13-15)^. A high prevalence of psychological illness was also observed, with many professionals developing symptoms of anxiety, depression, post-traumatic stress, and burnout due to their experiences during the pandemic^(10,16-18)^.

Some studies conducted during the COVID-19 pandemic theorized an increase in the clinical severity of patients with non-communicable chronic diseases, as many did not receive regular treatment due to isolation measures^(19-20)^. This phenomenon may generate additional strain on the healthcare system, impacting already affected professionals more severely, especially since there has been insufficient time for recovery.

Although the literature highlights many challenges faced by nursing professionals before and during the pandemic, there remains a gap in understanding the post-pandemic effects on the health of these workers. Thus, this study is justified by the need to identify sick leave absenteeism among Brazilian nursing professionals during the pre-pandemic, pandemic, and post-pandemic periods. Furthermore, it becomes essential to investigate the factors contributing to absenteeism in different hospital care settings to discuss potential interventions in these contexts.

From this perspective, the objective of this study was to identify the factors related to sick leave absenteeism among Brazilian nursing professionals before, during, and after the COVID-19 pandemic.

## Method

### Study design

This is a cross-sectional, retrospective research guided by the Strengthening the Reporting of Observational Studies in Epidemiology (STROBE) tool^(21)^.

### Setting

The study was conducted at a quaternary teaching hospital in southern Brazil. The institution is public, affiliated with a university, and serves the Unified Health System (*Sistema Único de Saúde*, SUS). During the COVID-19 pandemic, the institution became a reference center for high-complexity care for infected patients. Specifically, the study was conducted based on absence data from nursing professionals in the adult emergency department, adult Intensive Care Unit (ICU), clinical inpatient units, and surgical inpatient units.

### Period

Based on the total number of cases reported by the National Council of Health Secretaries (*Conselho Nacional de Secretários de Saúde*, CONASS), the periods were defined as “Before” the pandemic, from March 2019 to March 2020; “During”, from April 2020 to April 2021; and “After”, from May 2021 to May 2022^(22)^.

The “Before” period was defined as the year prior to the start of social isolation in the state of Rio Grande do Sul, spanning from March 2019 to March 2020. By the end of this period, the state had approximately 1,000 confirmed COVID-19 cases. The “During” period was defined from April 2020 to April 2021, encompassing the exponential increase in cases and deaths in the state. The “After” period was defined from May 2021 to May 2022. In this context, vaccine distribution for healthcare professionals had already been established in the state^(23)^, and there was a decline in the number of COVID-19-related deaths.

### Participants

The total available population included 1,455 nursing professionals (nurses and nursing technicians) employed in the investigated departments. From this group, a smaller sample was obtained based on absence records.

The inclusion criteria were as follows: nursing professionals (nurses and nursing technicians) of both genders who had at least one work absence due to illness or medical procedures between March 2019 and May 2022. Absences due to unreported illnesses were considered losses and were beyond control.

For the purposes of the analyses, individuals with multiple absences in each period, as well as those with absences in only one of the periods, were included. Thus, each period represents a different final population, accounting for all professionals and their absences within the described timeframe.

### Data sources and variables

Data collection was performed through a Query requested from the institution, which maintained the data in an institutional database. The data extracted from the Occupational Medicine Department (*Setor de Medicina Ocupacional*, SMO) and the Human Resources Coordination Office (*Coordenadoria de Gestão de Pessoas*, CGP) were provided to the authors in an anonymized format. The data were compiled and organized by the first author after being provided in raw form by the institution. Selected variables were tabulated, including gender, age, ethnicity, position/role, department, time at the institution, COVID-19 infection, period and duration of absence, reason for absence, and ICD-10^(24)^ (International Statistical Classification of Diseases and Related Health Problems). Each case was assigned a numerical code for the identification of anonymized records.

The reasons for sick leave absenteeism, categorized according to ICD-10, were subdivided into six categories: COVID-19, Musculoskeletal/Traumatology, Nonspecific, Psychosocial and Infections, and the category “Others,” which included all categories appearing with a frequency below 5%. These were: Gastrointestinal, Obstetrics/Urology/Gynecology, Dermatology, Ophthalmology, Cardiology, Otorhinolaryngology, Vaccination, Oncology, Breast Cancer, Pre- and Post-Surgical, Chronic Diseases, Neurology, Pulmonary, Metabolic/Hematology.

### Sample calculation

Considering a total available population of up to 1,455 professionals, a sample size of 732 nursing professionals (244 in each group) was estimated to detect significant differences in Y among groups A, B, and C, with means of 4.2, 5.6, and 4.2 u.m. (units of measurement - days), respectively. With an additional 10% added for potential losses, a minimum of 816 individuals was obtained. The calculation considered 90% power, a 5% significance level, and a standard deviation of 5 u.m. (days). This calculation was performed using the online version of the PSS Health tool with the assistance of a statistical professional^(25)^. A final sample of 839 professionals was achieved, meeting the minimum required number of participants.

### Quantitative variables and statistical analyses

The data were organized and transferred from Microsoft Office Excel^®^ spreadsheets to the Statistical Package for the Social Sciences^®^ (SPSS) version 26.0 for Windows^®^. Descriptive analysis of the results was performed using absolute and relative frequencies (n; %), as well as measures of central tendency (mean and median) and variability (standard deviation and range).

To verify the distribution of continuous variables, the Kolmogorov-Smirnov test and Friedman analysis of variance test were used.

Bivariate analysis between categorical variables was conducted using Pearson’s chi-square test. For continuous variables compared across three or more groups, a One-Way Analysis of Variance (ANOVA) was performed.

The absenteeism rate was calculated using a simplified version of the formula, which is used internationally^(26)^ and in Brazilian guidelines^(27)^. The formula is described as: “Absenteeism rate = Total working days of absence x 100 / Total working days in the period x Total employees in the department.” The absenteeism rate for each analyzed month, as well as the average absenteeism rate for each of the three periods, was calculated.

To compare absenteeism rates between departments and periods, Autocorrelation (ACF) and Partial Autocorrelation (PACF), tests, as well as the Durbin-Watson statistic were applied, with values obtained being close to or above 1.5, indicating no significant autocorrelation. Complementarily, the Kruskal-Wallis test was used to assess differences in the medians of absenteeism rates between departments, followed by Dunn’s multiple comparisons test to identify which departments showed significant differences from one another.

The strength of association between sociodemographic and occupational variables regarding the reasons for sick leave absenteeism (p<0.20) was analyzed using the Poisson Regression Model with Robust Variance. Multivariate analysis was represented by the Prevalence Ratio (PR) and 95% Confidence Intervals (CI). All analyses adopted a significance level of 5%.

### Bias

One potential bias in this manuscript is the likelihood of errors in data entry and tabulation during the analysis process. To prevent this, double-checking was performed with the support of two distinct researchers involved in the study. Additionally, the presence of illness reasons classified as nonspecific may make the data more susceptible to biases.

### Ethical aspects

The study was submitted and approved by the Ethics and Research Committee (*Comitê de Ética e Pesquisa*, CEP/UFRGS) under CAAE: 69221923.0.0000.5327. A commitment term for data usage was signed, and a waiver of the Free and Informed Consent Form (FICF) was accepted due to the anonymization of the collected data. Thus, the ethical principles established in Resolution No. 466/2012 of the National Health Council^(28)^ and the General Data Protection Law (*Lei Geral de Proteção de Dados*, LGPD), Law No. 13,709 dated August 2018^(29)^, were respected.

## Results

A total of 839 professionals experienced sickness-related absenteeism during the analyzed period. Of these, n=477 individuals had absences in the “before” pandemic period, n=665 during the “during” pandemic period, and n=699 in the “after” pandemic period.

From the sample, 7,375 sickness-related absences were identified between March 2019 and May 2022. Of these absences, 1,855 (25.2%) occurred in the pre-pandemic period, 2,551 (34.6%) during the pandemic period, and 2,969 (40.3%) in the post-pandemic period.

The overall results revealed a predominance of female professionals (79.6%; n=668), white ethnicity (84.5%; n=709), and aged 41 years or younger (31.8%; n=267). Regarding marital status, the majority of professionals were single (72.6%; n=609). In terms of occupation, nursing technicians accounted for the highest percentage of absences (76.2%; n=639), compared to nurses (27.4%; n=200).

The distribution of absences by department was as follows: Emergency Department (17.4%; n=146), ICU (31.3%; n=263), Clinical Inpatient Unit (27.5%; n=231), and Surgical Inpatient Unit (23.7%; n=199). The average number of days lost per nursing professional was 54.1 (SD=2.5).

The statistically significant factors associated with sickness-related absenteeism among nursing professionals were the number of days lost per professional (p<0.001) and age group (p=0.003). The other variables did not show statistically significant differences across the periods before, during, and after the COVID-19 pandemic, as presented in Table 1.

**Table 1 -:** Kolmogorov-Smirnov tests and Friedman Analysis of Variance applied to characterize the nursing professionals on leave before, during, and after the COVID-19 pandemic (n = 839). Porto Alegre, RS, Brazil, 2023-2024

Variables	Total n=839 ( *f* )	1st Period (Before) n=477 ( *f* )	2nd Period (During) n=665 ( *f* )	3rd Period (After) n=699 ( *f* )	p-value
Number of days lost per worker*	54.1 (σ=2.5)	15.05 ^†^ (σ=1.7)	20.2 ^†^ (σ=0.9)	18.8 ^†^ (σ=1.2)	<0.001
Gender					0.655
Female	668 (79.6)	387 (81.1)	525 (78.9)	560 (80.1)	
Male	171 (20.4)	90 ^†^ (18.9)	140 (21.1)	139 (19.9)	
Age group					0.003
<41 years old	267 (31.8)	112 ^†^ (21.0)	202 ^†^ (37.9)	219 ^†^ (41.1)	
42-46 years old	221 (26.3)	129 ^†^ (26.3)	174 ^†^ (35.5)	187 ^†^ (38.2)	
41-52 years old	180 (21.5)	113 ^†^ (27.6)	147 ^†^ (35.6)	149 ^†^ (36.4)	
≥53 years old	171 (20.4)	123 ^†^ (30.1)	142 ^†^ (34.7)	144 ^†^ (35.2)	
Ethnicity					0.977
White	709 (84.5)	409 (26.0)	570 (36.6)	592 (37.7)	
Black	100 (11.9)	54 (25.6)	74 (35.1)	83 (39.3)	
Brown	30 (3.6)	14 (23.7)	21 (35.6)	24 (40.7)	
Marital status					0.416
With a partner	230 (27.4)	151 (28.0)	192 (35.6)	197 (36.5)	
Without a partner	609 (72.6)	326 (25.1)	473 (36.4)	502 (38.6)	
Occupation					0.969
Nurse	200 (23.8)	111 (26.2)	151 (35.6)	162 (38.2)	
Nursing Technician	639 (76.2)	366 (76.7)	514 (36.3)	537 (37.9)	
Work Sector					0.234
Emergency	146 (17.4)	85 (26.3)	113 (35.0)	125 (38.7)	
ICU	263 (31.3)	116 (21.6)	209 (38.9)	212 (39.5)	
Clinical Inpatient Unit	231 (27.5)	148 (28.2)	184 (35.0)	193 (36.8)	
Surgical Inpatient Unit	199 (23.7)	128 (28.1)	159 (34.9)	169 (37.1)	

*Mean and standard deviation; ^†^Represents subgroups that differ from each other at p-value<0.05

Before the pandemic, the highest absenteeism rates in the sectors were 4.1% in the Emergency Department in March 2020, 7.5% in the ICU in January 2020, 6.2% in the Clinical Inpatient Unit in October 2019, and 5% in the Surgical Inpatient Unit in March 2020. During the pandemic, the Emergency Department saw an increase, reaching 8.2% in April 2021. The Clinical Inpatient Unit peaked at 9.9% in July 2020; however, the ICU showed a reduction, with its highest rate being 4.3% in June 2020. In contrast, the Surgical Inpatient Unit reached 9.3% in June 2020. In the post-pandemic period, the Emergency Department recorded 8.2% in August 2021, the ICU reached 5% in April 2022, the Clinical Inpatient Unit remained elevated at 7.8% in May 2022, and the highest absenteeism rate in the Surgical Inpatient Unit was 6.6% in June 2021.

**Figure 1 -:**
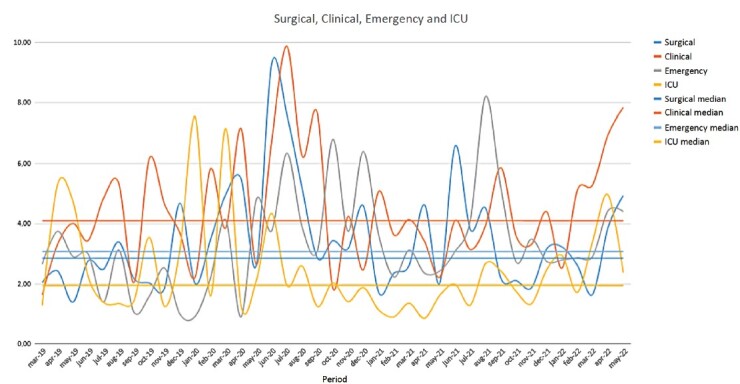
Sickness-related absenteeism rate according to the work sectors of nursing professionals on leave before, during, and after the COVID-19 pandemic (n = 839). Porto Alegre, RS, Brazil, 2023-2024

Autocorrelation (ACF) and Partial Autocorrelation (PACF) charts, along with Durbin-Watson statistics, indicated no evidence of autocorrelation in the absenteeism series or in the residuals of regressions involving the work sectors of nursing professionals. Additionally, the Kruskal-Wallis analysis revealed significant differences in the median monthly absenteeism rates among the four evaluated sectors (Emergency, ICU, Clinical Inpatient Unit, and Surgical Inpatient Unit). Specifically, Dunn’s multiple comparisons test showed that the median monthly sickness-related absenteeism in the ICU was significantly different compared to the other sectors (Figure 1).

In Table 2, the main reasons for sickness-related absenteeism among nursing professionals are presented, categorized according to the analyzed periods. From the data analysis, six primary reasons were identified: musculoskeletal disorders, psychosocial conditions, pulmonary infections (excluding COVID-19), nonspecific conditions, COVID-19 infection, and a broad category termed “Others”. The “Others” category includes all conditions with a frequency below 5%, such as Gastrointestinal, Obstetrics/Urology/Gynecology, Dermatology, Ophthalmology, Cardiology, Otorhinolaryngology, Vaccination, Oncology, Breast Cancer, Pre- and Post-Surgical, Chronic Diseases, Neurology, Pulmonary, and Metabolic/Hematology. All reasons were classified according to the International Classification of Diseases (ICD-10) and organized chronologically across the pre-pandemic, during-pandemic, and post-pandemic periods of COVID-19.

**Table 2 -:** Pearson’s Chi-square tests and One-Way Analysis of Variance (ANOVA) applied to the reasons for sickness-related absenteeism among nursing professionals according to the periods (n = 839). Porto Alegre, RS, Brazil, 2023-2024

Reasons for sickness-related absenteeism	1st Period (Before) n*= 477 ( *f* % ^†^ )	2nd Period (During) n*= 665 ( *f* % ^†^ )	3rd Period (After) n*= 699 ( *f* % ^†^ )	p ^‡^
COVID-19 (Yes)	34 ^§^ (7.1)	385 ^§^ (42.1)	275 ^§^ (39.3)	<0.001
(No)	443 (92.9)	280 (57.9)	424 (60.7)	
Musculoskeletal (Yes)	238 ^§^ (49.9)	292 ^§^ (43.9)	350 ^§^ (50.1)	<0.001
(No)	239 (50.1)	373 (56.1)	349 (49.9)	
Psychosocial (Yes)	111 (23.3)	123 (18.5)	125 (17.9)	0.263
(No)	366 (76.6)	542 (81.5)	574 (82.1)	
Pulmonary infectious (Yes)	129 ^§^ (27)	170 ^§^ (25.6)	249 ^§^ (35.6)	0.003
(No)	348 (73)	495 (74.4)	450 (64.4)	
Unspecified conditions (Yes)	268 ^§^ (43.8)	271 ^§^ (40.8)	321 ^§^ (45.9)	0.001
(No)	209 (56.2)	394 (59.28)	378 (54.1)	
Others (Yes)	222 ^§^ (46.5)	187 ^§^ (28.1)	264 ^§^ (37.8)	<0.001
(No)	225 (53.5)	478 (71.9)	435 (62.5)	

*n = Total sample; ^†^ *f*% = Sample percentage; ^‡^p = p-value; ^§^Represents subgroups that differ from each other at p-value <0.05

Regarding the factors contributing to illness among nursing professionals, Table 3 presents significant associations of sickness-related absenteeism reasons based on the Poisson Regression Model with robust variance, across the periods before, during, and after the COVID-19 pandemic.

**Table 3 -:** Poisson regression model with robust variance for variables associated with sickness-related absenteeism reasons among nursing professionals in the periods before, during, and after the COVID-19 pandemic (n = 839). Porto Alegre, RS, Brazil, 2023-2024

T*	Variables	COVID-19		Musculoskeletal		Psychosocial		Infectious		Unspecified		Others	
		OR ^†^ (95% CI ^‡^ )	p ^§^	OR ^†^ (95% CI ^‡^ )	p ^§^	OR ^†^ (95% CI ^‡^ )	p ^§^	OR ^†^ (95% CI ^‡^ )	p ^§^	OR ^†^ (95% CI ^‡^ )	p ^§^	OR ^†^ (95% CI ^‡^ )	p ^§^
**1st**	**Gender**												
	Female									1			
	Male									0.234 (0.124-0.440)	0.001	0.342 (0.180-0.648)	0.001
	**Age (years old)**												
	<41			1						1			
	42-46			1.242 (0.987-1.824)	0.06					0.604 (0.274-1.329)	0.21		
	47-52			1.332 (0.969-1.829)	0.077					1.477 (0.593-3.678)	0.402		
	≥53			1.425 (1.036-1.961)	0.029					4.974 (1.502-16.47)	0.009		
	**Occupation**												
	Nurse							1					
	Nursing Technician							1.900 (1.187-3.040)	0.007				
	**Sector**												
	Emergency			1		1		1		1			
	Intensive Care			0.677 (0.486-0.944)	0.021	0.415 (0.229-0.752)	0.004	0.547 (0.328-0.914)	0.021	1.974 (0.631-6.174)	0.242		
	Clinical Hospitalization			1.036 (0.766-1.401)	0.82	0.837 (0.517-1.355)	0.47	1.197 (0.776-1.845)	0.416	3.482 (1.244-9.747)	0.017		
	Surgical Hospitalization			1.007 (0.738-1.373)	0.966	1.153 (0.726-1.830)	0.546	0.848 (0.523-1.376)	0.505	1.198 (0.533-2.691)	0.662		
**2nd**	**Marital status**												
	With a partner							1					
	Without a partner							0.643 (0.489-0.845)	0.002				
	**Occupation**												
	Nurse			1									
	Nursing Technician			1.341 (1.043-1.724)	0.022								
**3rd**	**Gender**												
	Female									1			
	Male									0.536 (0.339-0.848)	0.008		
	**Marital status**												
	With a partner					1							
	Without a partner					0.691 (0.495-0.965)	0.03						
	**Occupation**												
	Nurse			1		1							
	Nursing Technician			1.456 (1.165-1.820)	0.001	1.756 (1.112-2.772)	0.016						
	**Sector**												
	Emergency	1		1									
	Intensive Care	0.784 (0.608-1.011)	0.06	0.747 (0.593-0.941)	0.013								
	Clinical Hospitalization	0.655 (0.496-0.865)	0.003	0.866 (0.691-1.086)	0.213								
	Surgical Hospitalization	0.701 (0528-0.930)	0.014	0.891 (0.709-1.820)	0.321								

*T = Time period; ^†^PR = Prevalence Ratio; ^‡^CI = Confidence Interval; ^§^p = p-value

## Discussion

This study identified and analyzed factors related to sickness-related absenteeism among Brazilian nursing professionals before, during, and after the COVID-19 pandemic. It was evident that sociodemographic factors such as age group and the number of days lost are associated with absenteeism among these professionals. Absenteeism rates increased significantly during the pandemic, and the ICU sector showed a significantly different median monthly sickness-related absenteeism rate compared to other sectors. The most prevalent causes of illness were musculoskeletal disorders, COVID-19, other pulmonary infections, and unspecified conditions.

This study identified an increase in absenteeism rates post-pandemic, surpassing those recorded in the pre-pandemic period—although rates were considered high across all periods. Some international studies have already reported a rapid rise in absenteeism and illness among professionals during the pandemic^(15,30-31)^. However, there are still no definitive studies on sickness-related absenteeism post-pandemic. It can be assumed that the elevated post-pandemic absenteeism rates represent possible health sequelae among nursing professionals, who were already affected by occupational illnesses during the pandemic.

The highest rates were concentrated during the pandemic in 2020, with 9.3% in June in the Surgical Inpatient Unit and 9.9% in July in the Clinical Inpatient Unit. These figures were significantly higher than the expected limit of 6.7% for unplanned absences established by the Federal Nursing Council (COFEN), particularly during the pandemic period, underscoring the significant impact of the pandemic on nursing during this time^(32)^. Another study diverged from these findings, identifying that the lowest monthly absenteeism rate occurred in the pre-pandemic period, at 2.07% in December 2019, while the highest rate was 9.82% in July 2020^(33)^.

In the pre-pandemic period, the predominant age group was 53 years or older, representing 123 cases (30.1%), the highest percentage recorded during this period. Conversely, during the pandemic, the most prevalent age group was 41 years old or younger, totaling 202 cases (37.9%), which remained consistent in the post-pandemic period, with 219 cases (41.1%). Aligning with these data, some studies conducted during the pandemic demonstrated that the nursing staff, in association with the work environment, predominantly fell within the 36–40-year age group, showing a significant relationship with absenteeism^(10,34)^. It is worth noting that during the pandemic, guidelines recommended that individuals belonging to risk groups refrain from their work activities, typically including older individuals. A multicenter Brazilian study reinforces that age is a significant risk factor among active professionals during the pandemic. Due to the risk of contracting the virus, professionals in risk groups expressed concerns about their work activities, which could lead to psychological harm^(35)^.

In the pre-pandemic period, a study conducted in Chile demonstrated that physical fatigue increased the likelihood of workplace absenteeism by 1.05 times. Additionally, working for more than one year in the same clinical service increased the risk of absenteeism by 1.084 times^(36)^. Another study analyzing 2,761 nursing professional absences revealed that 449 (16.26%) were related to musculoskeletal disorders. In this study, the service with the most absences was clinical inpatient care, and the group with the longest absences (>15 days) consisted of nursing assistants and technicians (p=0.006), workers with a lower median age (p=0.021), and higher education levels (p=0.035)^(37)^. Moreover, being a professional in the Clinical Inpatient Unit demonstrated a 3.48 times higher probability of absences due to unspecified conditions compared to the Emergency Department, which had the lowest prevalence.

During the pandemic, the strongest observed association was related to occupation, where nursing technicians had a 1.34 times higher probability of absenteeism due to musculoskeletal diseases compared to nurses. Consistent with these results, other studies indicated that nursing technicians exhibited a higher rate of absences in emergency and urgent care units^(34)^ as well as in wards^(38)^. A study conducted in Ecuador found that 85% of nursing assistants suffer from osteomyoarticular diseases, with a higher incidence in the lumbosacral region and lower limbs during their work shifts. Furthermore, it was observed that the high prevalence of these health issues is directly related to increased absenteeism in ward units. It was also identified that 39% of participants required temporary leave from work for one to three days due to their health conditions^(38)^.

Contrary to the lower percentages of sickness-related absenteeism in the Emergency Department in this study, other authors highlighted the context in which these professionals operated, characterized by its emergency, variable, and unpredictable nature, requiring a high workload and the management of complex cases^(8)^. This demands professional engagement in labor activities encompassing physical, mental, and psychosocial aspects, given the range of vulnerabilities associated with their work environment. It is worth noting that when absenteeism rates among nursing professionals are high, it becomes more complex to adjust work schedules to meet all demands required by the workload.

Although this research did not find significant associations between sickness-related absenteeism and psychosocial factors or psychosomatic illnesses, some studies have highlighted relevant impacts on the mental health of healthcare professionals.

A Canadian study linked psychological impacts to a combination of factors, both personal life and workplace burdens, specifically in intensive care settings. In this context, nurses experienced significant psychological distress during the COVID-19 pandemic^(11)^.

In Atlanta, ICU nurses, an environment where Burnout Syndrome was already common among the multidisciplinary team before the COVID-19 pandemic, saw a substantial increase in burnout prevalence during the pandemic^(39)^. A similar situation was observed among ICU professionals in Brazil, where high levels of emotional exhaustion and depersonalization were already present prior to the pandemic due to excessive workloads^(40)^.

A survey conducted with mental health institution professionals in the Netherlands between 2021 and 2022 revealed that most respondents reported neither an increase nor a decrease in symptoms of anxiety, depression, stress, sadness, and/or anger. However, 35.7% (n=182) of respondents reported “more symptoms” of stress, and 20.6% (n=105) noted an increase in depressive symptoms compared to the period during the pandemic. These findings indicate that such symptoms were significantly more intense and prevalent during the pandemic than in the post-pandemic period. Additionally, an increase in sickness-related absenteeism was observed in the post-pandemic period, accompanied by a higher frequency of absences, results that align with those found in this study^(41)^.

In Jordan, nurses working in clinical/surgical units and intensive care units reported significantly higher levels of job satisfaction, with lower absence rates and intentions to leave their jobs during the pandemic compared to the pre-COVID-19 period^(42)^. A multicenter study conducted with ICU nursing workers at four hospitals designated for COVID-19 care revealed that resilience positively influenced the domains of emotional exhaustion and low professional achievement associated with Burnout Syndrome. The study also highlighted that the level of exposure to COVID-19 significantly impacted professionals’ perceptions of the pandemic’s effects on their mental health^(43)^.

According to other studies, the main causes of absenteeism during the pandemic were related to COVID-19 infection, respiratory issues, musculoskeletal conditions, family matters, health satisfaction, and mental health problems^(7,44-45)^, with particular emphasis on anxiety, depression, and stress^(10,18)^. An integrative review revealed that 92% of the analyzed articles cited musculoskeletal system diseases, 64% reported mental and behavioral/psychological disorders, and 48% mentioned respiratory system diseases as the most prevalent^(4)^. Additionally, one study identified that the inexperience of nursing professionals in COVID-19-dedicated units represented an increased burden for experienced professionals, who had to conduct training and supervision sessions. Despite this, it was confirmed that these professionals, even working in different sectors with varying levels of workplace exposure, faced similar health repercussions^(46)^.

It is important to consider some limitations when interpreting the results of this study. Firstly, the impossibility of generalizing the results to other areas and institutions should be noted, as the research was conducted in hospital environments within a single institution, which has unique characteristics. Moreover, the presence of unspecified illness causes may make the data more susceptible to biases.

Finally, these findings allow technicians and nurses to recognize the factors influencing absenteeism in their daily work routines, encouraging reflection and prompting actions aimed at preventing illness.

## Conclusion

The factors associated with sickness-related absenteeism among Brazilian nursing professionals before, during and after the COVID-19 pandemic were statistically linked to the overall mean number of leave days per professional and the age group of 41 years old or younger. In other words, professionals with longer leave periods, as well as those in the young-adult phase, were more likely to miss work. In addition, the mean number of lost days was significantly higher during the pandemic.

Absence rates during and after the pandemic exceeded those recorded in the pre-pandemic period, although they remained consistently high across all analyzed periods. Among the main reasons for sickness-related absenteeism were COVID-19 infection and musculoskeletal diseases.

The identification of aspects related to sickness-related absenteeism among Brazilian nursing professionals provides a significant contribution to the academic debate on the pandemic’s impacts on occupational health, especially within an essential professional category like nursing. The results of this study offer valuable insights for managers to develop more effective preventive and corrective policies targeting professionals with multiple absences or extended periods of absence. On a social level, the importance of prioritizing the physical and mental health of these professionals is emphasized, considering the risks and challenges inherent to their work, particularly in crisis contexts such as pandemics.

Thus, this study reinforces the need to implement integrated strategies that promote the well-being of these professionals, ensuring the sustainability of the healthcare system and the quality of care provided to the population.
