# Supplementary material for: Factors related to sickness absenteeism among Brazilian Nursing professionals before, during, and after the pandemic
Source: Rev Lat Am Enfermagem. 2025 Jul 28;33:e4623. doi: 10.1590/1518-8345.7696.4623 (PMC12306928; doi:10.1590/1518-8345.7696.4623)
Supplement: Translation

# Translation: Fatores relacionados ao absenteísmo-doença entre profissionais de enfermagem brasileiros antes, durante e após a pandemia*

**DOI:** 10.1590/1518-8345.7696.4624

**Authors:** Fernanda Seidel Pinheiro, Miguel Lucas Silva da Paixão, Gabriel Fernandes Gonçalves, João Lucas Campos de Oliveira, Daiane Dal Pai, Juliana Petri Tavares

**Affiliations:** 1 Universidade Federal do Rio Grande do Sul, Escola de Enfermagem, Porto Alegre, RS, Brasil.

**Keywords:** Absenteísmo, Saúde do Trabalhador, Enfermagem, COVID-19, Pandemias, Gestão de Recursos Humanos

## Abstract

identificar os fatores relacionados ao absenteísmo-doença de profissionais de Enfermagem brasileiros antes, durante e após a pandemia da COVID-19.

estudo transversal com profissionais de Enfermagem de internação clínica, cirúrgica, centro de tratamento intensivo e emergência adulta, com afastamentos entre 2019-2022. Avaliaram-se as variáveis sociodemográficas, laborais e de afastamento. Análise estatística descritiva, cálculo de taxa de absenteísmo e Regressão de Poisson com variância robusta, considerando p≤0,05.

amostra de 839 profissionais, com 7.375 afastamentos. Absenteísmo-doença levou a uma média de 54,1±2,5 dias perdidos (p<0,001) e a profissionais com 41 anos ou menos (31,8%; p=0,003). Setores terapia intensiva (31,3%) e internação clínica (27,5%) apresentaram mais afastamentos. Maior taxa de absenteísmo (9,9%) em julho/2020. O risco de adoecimento esteve relacionado ao sexo masculino (p≤0,001) e terapia intensiva (p=0,007) no 1º período; solteiros (p=0,002) e técnicos de Enfermagem (p=0,022) no 2º período; e terapia intensiva (p=0,003) e técnicos de Enfermagem (p≤0,001) no 3º período.

após o fim da pandemia, as taxas de absenteísmo não retornaram aos parâmetros pré-pandemia. COVID-19 e doenças osteomusculares foram mais prevalentes. Foi possível investigar os fatores relacionados ao absenteísmo.

## Destaques

**(1)** Número de dias ausentes e idade ≤ 41 anos estão associados ao absenteísmo. **(2)** As principais causas de absenteísmo-doença foram COVID-19 e doenças osteomusculares. **(3)** Internação clínica teve a maior taxa de absenteísmo entre todos os setores e períodos. **(4)** Absenteísmo no 2º e 3º período superou o 1º, mas foi alto em todos os períodos.

## Introdução

O adoecimento laboral é uma discussão antiga, mas ainda é um problema persistente, inclusive no que tange aos profissionais de Enfermagem. O absenteísmo entre os profissionais de Enfermagem constitui uma questão de grande relevância para a saúde pública, e afeta diretamente a qualidade do atendimento e a sustentabilidade dos serviços de saúde. Sabe-se que no absenteísmo-doença estão incluídas todas as faltas por motivo de doença ou procedimento médico, com exceção das doenças ocupacionais^(1)^. Quando ocorre entre os trabalhadores de Enfermagem, o absenteísmo desorganiza o serviço, provoca insatisfação e sobrecarga na equipe, resultando na diminuição da qualidade da assistência prestada aos pacientes^(2)^.

Os impactos psicossociais e psicossomáticos decorrentes da natureza do trabalho na Enfermagem reduzem a produtividade e tendem a aumentar os traumas, a exaustão emocional, o medo de se contaminar, além de sentimentos como: tristeza, mau humor e desejo de desistir de tudo, contribuindo para o aumento do absenteísmo desses profissionais nos serviços de saúde^(3-6)^.

Entre as principais causas de absenteísmo na Enfermagem, estão as doenças do aparelho respiratório, as doenças infecciosas e parasitárias, além de doenças do sistema osteomuscular e do tecido conjuntivo, frequentemente resultantes das exposições ocupacionais^(7)^. Destacam-se, em particular, as doenças mentais e comportamentais, clínicas, osteomusculares e de tecido conjuntivo, que geralmente apresentam os percentuais mais elevados^(5,8)^.

Com o advento da pandemia da COVID-19, maximizaram-se os problemas que afetaram diariamente os profissionais de Enfermagem, como a sobrecarga de trabalho, jornadas de trabalho extensas e exaustivas, má qualidade do sono, dupla jornada de trabalho, processos de trabalho e recursos materiais ineficazes, entre outros^(9-10)^. Frente à rápida disseminação da COVID-19, estudos nacionais e internacionais relataram preocupações crescentes, incluindo a antecipação do impacto da doença, temor pela diminuição ou falta de equipamentos de proteção individual (EPI), além do medo pela própria segurança e dos entes queridos, que eram alarmantes^(11-12)^.

Internacionalmente, alguns estudos identificaram um risco aumentado de morbidade física grave e longos períodos de afastamento para os profissionais de Enfermagem que atenderam os pacientes com COVID-19^(13-15)^. Observou-se, também, uma elevada prevalência de adoecimento psicológico, com diversos profissionais desenvolvendo sintomas de ansiedade, depressão, estresse pós-traumático e *burnout* devido às experiências vivenciadas durante a pandemia^(10,16-18)^.

Alguns estudos realizados durante a pandemia da COVID-19 teorizaram um aumento na gravidade clínica dos pacientes com doenças crônicas não transmissíveis, uma vez que muitos não receberam tratamento regular devido ao isolamento^(19-20)^. Este fenômeno pode gerar uma nova sobrecarga no sistema de saúde, impactando de forma mais grave os profissionais já adoecidos pelos acontecimentos da pandemia, especialmente porque não houve tempo suficiente para sua recuperação.

Embora a literatura apresente muitos dos desafios enfrentados pela Enfermagem antes e durante a pandemia, ainda há uma lacuna no conhecimento sobre os efeitos pós-pandemia na saúde desses trabalhadores. Assim, este estudo justifica-se pela necessidade de identificar o absenteísmo-doença dos profissionais de Enfermagem brasileiros nos períodos antes, durante e após a pandemia da COVID-19. Ademais, torna-se essencial investigar os fatores que contribuíram para o absenteísmo destes profissionais nos diferentes setores de atendimento hospitalar, a fim de discutir as ações que possam ser implementadas nesses contextos.

Nesta perspectiva, o objetivo deste estudo foi identificar os fatores relacionados ao absenteísmo-doença de profissionais de Enfermagem brasileiros antes, durante e após a pandemia da COVID-19.

## Método

### Delineamento do estudo

Trata-se de uma pesquisa do tipo transversal, retrospectiva, norteada pela ferramenta *Strengthening the Reporting of Observational Studies in Epidemiology* (STROBE)^(21)^.

### Cenário

O estudo foi realizado em um hospital-escola quaternário, do sul do Brasil. A instituição é pública, vinculada a uma universidade e atende ao Sistema Único de Saúde (SUS). Durante a pandemia pela COVID-19, a instituição estabeleceu-se como referência no atendimento de alta complexidade aos pacientes infectados. Precisamente, o estudo ocorreu com base em dados de afastamentos dos profissionais de Enfermagem dos setores Emergência para adultos, Centro de Tratamento Intensivo (CTI) adulto, Unidades de Internação Clínica e Unidades de Internação Cirúrgica.

### Período

A partir dos números de casos informados na íntegra pelo Conselho Nacional de Secretários de Saúde (CONASS), foram denominados como períodos o “Antes” da pandemia, de março de 2019 a março de 2020, “Durante”, de abril de 2020 a abril de 2021, e “Após”, de maio de 2021 a maio de 2022^(22)^.

O período “Antes” foi definido como o espaço de um ano antes do início do isolamento social no estado do Rio Grande do Sul, de março de 2019 a março de 2020. Ao final deste período, o estado contava com aproximadamente 1.000 casos confirmados de COVID-19. O período “Durante” foi definido de abril de 2020 até abril de 2021, contemplando o aumento exponencial de casos e óbitos no estado. O período “Após” foi definido de maio de 2021 a maio de 2022. Nesse contexto, a distribuição de vacinas para os profissionais da saúde já havia sido estabelecida no estado^(23)^, e houve um declínio no número de óbitos pela COVID-19.

### Participantes

A população total disponível incluiu os 1.455 profissionais de Enfermagem (enfermeiros e técnicos de Enfermagem) contratados pelos setores investigados. Destes, um total reduzido foi obtido por meio dos dados de afastamentos.

Os critérios de inclusão foram: profissionais de Enfermagem (enfermeiros e técnicos de Enfermagem), de ambos os sexos, e que apresentaram pelo menos um afastamento do trabalho por doença ou procedimento médico entre março de 2019 e maio de 2022. As faltas motivadas por doenças não registradas foram consideradas perdas, sem controle.

Para fins das análises executadas, foram incluídos os indivíduos com múltiplos afastamentos em cada período, bem como os indivíduos com afastamentos em apenas um dos períodos. Dessa forma, cada um dos períodos apresenta uma população final diferente, representando todos os profissionais e seus afastamentos dentro do tempo descrito.

### Fontes de dados e variáveis

A coleta de dados foi realizada por meio de *Query* solicitada à instituição, que já os mantinha em banco institucional. Os dados extraídos do Setor de Medicina Ocupacional (SMO) e da Coordenadoria de Gestão de Pessoas (CGP) foram disponibilizados aos autores de forma anonimizada. Os dados foram compilados e organizados pela primeira autora, após serem fornecidos em versão bruta pela instituição. Foram tabeladas as variáveis selecionadas, incluindo sexo, idade, etnia, cargo/função, setor de atuação, tempo na instituição, infecção pela Covid-19, período e duração do afastamento, motivo do afastamento e CID-10^(24)^ (Classificação Estatística Internacional de Doenças e Problemas Relacionados com a Saúde). Cada caso recebeu um código numérico para a identificação dos prontuários anonimizados.

Os motivos do absenteísmo-doença, categorizados conforme CID-10, foram subdivididos em seis categorias: COVID-19, Osteomuscular/Traumatologia, Inespecíficas, Psicossocial e Infecções, e a categoria Outros, constituída por todas as categorias que apareceram com uma frequência inferior a 5%, sendo estas: Gastrointestinal, Obstetrícia/Urologia/Ginecologia, Dermatologia, Oftalmologia, Cardiologia, Otorrinolaringologia, Vacinação, Oncologia, Câncer de Mama, Pré- e Pós-Operatório, Doenças Crônicas, Neurologia, Pulmonar, Metabólica/Hematologia.

### Cálculo amostral

Ao se considerar um quadro total disponível de até 1.455 profissionais, foi estimado um tamanho de amostra de 732 profissionais de Enfermagem (244 em cada grupo) para detectar as diferenças significativas de Y entre os grupos A, B e C sendo 4.2, 5.6 e 4,2 u.m. (unidades de medida - dias) as médias consideradas para cada grupo, respectivamente. Com o acréscimo de 10% para as possíveis perdas, obteve-se o número mínimo de 816 indivíduos. O cálculo considerou poder de 90%, nível de significância de 5% e desvio-padrão igual a 5 u.m. (dias). Este cálculo foi realizado por meio da ferramenta *PSS Health* versão *online* com o auxílio de um profissional estatístico^(25)^. Considerou-se a amostra final de 839 profissionais, e atingiu-se o mínimo necessário de indivíduos.

### Variáveis quantitativas e análises estatísticas

Os dados foram organizados e transportados da planilha do *Microsoft OfficeExcel* ^®^ para o programa *Statistical Package for the Social Sciences* ^®^ (SPSS) versão 26.0 para *Windows* ^®^. A análise descritiva dos resultados ocorreu por frequência absoluta e relativa (n;%), assim como pelas medidas de tendência central (média e mediana) e de variabilidade (desvio-padrão e amplitude).

Para verificar a distribuição das variáveis contínuas, foi utilizado o teste de Kolmogorov-Smirnov e o teste de análise de variância de Friedman.

A análise bivariada entre as variáveis categóricas se deu pelo teste de Qui-quadrado de Pearson. Para as variáveis contínuas comparadas entre três ou mais grupos, foi realizado o teste de Análise de Variância (*One-Way*).

A taxa de absenteísmo foi obtida por meio do número de dias perdidos, com uma versão simplificada da fórmula, que é utilizada internacionalmente^(26)^ e em diretrizes brasileiras^(27)^. A fórmula é descrita como: “Taxa de absenteísmo = Total de dias úteis de ausência x 100 / Total de dias úteis no período x Total de funcionários no setor”. Calculou-se a taxa de absenteísmo de cada mês analisado, bem como a taxa de absenteísmo média de cada um dos três períodos.

Para comparar as taxas de absenteísmo entre setores e períodos, foram aplicados os testes de Autocorrelação (ACF), Autocorrelação Parcial (PACF) e a estatística de Durbin-Watson, cujos valores obtidos foram próximos ou superiores a 1,5, indicando a ausência de autocorrelação significativa. Complementarmente, utilizou-se o teste de Kruskal-Wallis para avaliar as diferenças nas medianas das taxas de absenteísmo entre os setores, seguido pelo teste de comparações múltiplas de Dunn para identificar quais setores apresentaram diferenças significativas entre si.

A análise da força de associação das variáveis sociodemográficas e ocupacionais em relação aos motivos de absenteísmo-doença (p<0,20) foi realizada por meio do Modelo de Regressão de Poisson com Variância Robusta, cuja análise multivariada foi representada pela Razão de Prevalência (RP) e Intervalos de Confiança (IC 95%). Para critérios de decisão estatística, todas as análises adotaram o nível de significância de 5%.

### Viés

Um dos possíveis vieses deste manuscrito é a chance de erro na digitação e na tabulação dos dados durante a realização das análises. Para evitar o mesmo, foi realizada a dupla conferência com o apoio de dois pesquisadores distintos envolvidos no estudo. Adicionalmente, a presença dos motivos de adoecimento classificados como inespecíficos pode tornar os dados mais suscetíveis a vieses.

### Aspectos éticos

O estudo foi submetido e aprovado pelo Comitê de Ética e Pesquisa (CEP/UFRGS), sob o CAAE: 69221923.0.0000.5327. Foi assinado o termo de compromisso com a utilização dos dados e foi aceita a dispensa do Termo de Consentimento Livre e Esclarecido (TCLE), devido à anonimização dos dados coletados. Portanto, foram respeitados os princípios éticos estabelecidos na Resolução nº 466/2012 do Conselho Nacional de Saúde^(28)^ e a Lei Geral de Proteção de Dados (LGPD), Lei nº 13.709, de agosto de 2018^(29)^.

## Resultados

Um total 839 profissionais apresentaram absenteísmo-doença no período analisado. Destes, n=477 indivíduos apresentaram afastamentos no período “antes” da pandemia, n=665 no período “durante”, e n=699 no período “após”.

A partir da amostra, identificaram-se 7.375 afastamentos por absenteísmo-doença entre o período de março de 2019 a maio de 2022. Destes afastamentos, 1.855 (25,2%) ocorreram no período antes da pandemia; 2.551 (34,6%) no período durante a pandemia; e 2.969 (40,3%) no pós-pandemia.

Os resultados gerais identificaram uma predominância de profissionais do sexo feminino (79,6%; n=668), de etnia branca (84,5%; n=709) e com idade igual ou inferior a 41 anos, com (31,8%; n=267). Quanto ao estado civil, a maioria dos profissionais estava sem parceiros (72,6%; n=609). Em relação à ocupação, os técnicos de Enfermagem representavam o maior percentual de afastamentos (76,2%; n=639), comparado aos enfermeiros (27,4%; n=200).

Os profissionais afastados por setor de atuação distribuíram-se da seguinte forma: Emergência (17,4%; n=146), CTI (31,3%; n=263), Unidade de Internação Clínica (27,5%; n=231), e Unidade de Internação Cirúrgica (23,7%; n=199). A média do número de dias perdidos por profissional de Enfermagem foi de 54,1 (dp=2,5)

Os fatores estatisticamente associados ao absenteísmo-doença dos profissionais de Enfermagem foram o número de dias perdidos por profissional (p<0,001) e a faixa etária (p=0,003). As demais variáveis não apresentaram diferença estatisticamente significativa nos períodos, antes, durante e após a pandemia pela COVID-19, conforme apresentado na Tabela 1.

**Tabela 1 -:** Testes de Kolmogorov-Smirnov e Análise de Variância de Friedman aplicados à caracterização dos profissionais de Enfermagem afastados, antes, durante e após a pandemia de COVID-19 (n = 839). Porto Alegre, RS, Brasil, 2023-2024

**Variáveis**	**Total n=839 ( *f* )**	**1º Período (Antes) n=477 ( *f* )**	**2º Período (Durante) n=665 ( *f* )**	**3º Período (Após) n=699 ( *f* )**	**p-valor**
Nº de dias perdidos por profissional*	54,1 (σ=2,5)	15,05 ^†^ (σ=1,7)	20,2 ^†^ (σ=0,9)	18,8 ^†^ (σ=1,2)	<0,001
Sexo					0,655
Feminino	668 (79,6)	387 (81,1)	525 (78,9)	560 (80,1)	
Masculino	171 (20,4)	90 ^†^ (18,9)	140 (21,1)	139 (19,9)	
Faixa etária					0,003
< 41 anos	267 (31,8)	112 ^†^ (21,0)	202 ^†^ (37,9)	219 ^†^ (41,1)	
42 a 46 anos	221 (26,3)	129 ^†^ (26,3)	174 ^†^ (35,5)	187 ^†^ (38,2)	
47 a 52 anos	180 (21,5)	113 ^†^ (27,6)	147 ^†^ (35,6)	149 ^†^ (36,4)	
≥ 53 anos	171 (20,4)	123 ^†^ (30,1)	142 ^†^ (34,7)	144 ^†^ (35,2)	
Etnia					0,977
Branco	709 (84,5)	409 (26,0)	570 (36,6)	592 (37,7)	
Preto	100 (11,9)	54 (25,6)	74 (35,1)	83 (39,3)	
Pardo	30 (3,6)	14 (23,7)	21 (35,6)	24 (40,7)	
Estado civil					0,416
Com parceiro	230 (27,4)	151 (28,0)	192 (35,6)	197 (36,5)	
Sem parceiro	609 (72,6)	326 (25,1)	473 (36,4)	502 (38,6)	
Ocupação					0,969
Enfermeiro(a)	200 (23,8)	111 (26,2)	151 (35,6)	162 (38,2)	
Técnico(a) de Enfermagem	639 (76,2)	366 (76,7)	514 (36,3)	537 (37,9)	
Setor de atuação					0,234
Emergência	146 (17,4)	85 (26,3)	113 (35,0)	125 (38,7)	
CTI	263 (31,3)	116 (21,6)	209 (38,9)	212 (39,5)	
Internação Clínica	231 (27,5)	148 (28,2)	184 (35,0)	193 (36,8)	
Internação Cirúrgica	199 (23,7)	128 (28,1)	159 (34,9)	169 (37,1)	

*Média e desvio-padrão; ^†^Representam subgrupos que diferem entre si a um valor de p<0,05

Antes da pandemia, as maiores taxas de absenteísmo nos setores foram de 4,1% na Emergência em março de 2020, 7,5% no CTI, em janeiro de 2020, 6,2% na Unidade de Internação Clínica, em outubro de 2019 e 5% na Unidade de Internação Cirúrgica, em março de 2020. Durante a pandemia, a Emergência registrou um aumento, chegando a 8,2% em abril de 2021. A Unidade de Internação Clínica atingiu 9,9% em julho de 2020, porém, o CTI apresentou uma redução, sendo sua maior taxa 4,3%, em junho de 2020. Em contraste, a Unidade de Internação Cirúrgica alcançou 9,3%, em junho de 2020. No período pós-pandemia, a Emergência registrou 8,2%, em agosto de 2021, o CTI apresentou 5%, em abril de 2022, a Internação Clínica manteve-se elevada com 7,8%, em maio de 2022, e a maior taxa de absenteísmo na Unidade de Internação Cirúrgica foi de 6,6%, em junho de 2021.

Os Gráficos de Autocorrelação (ACF) e Autocorrelação Parcial (PACF), juntamente com as estatísticas de Durbin-Watson, indicaram que não há evidência de autocorrelação nas séries de absenteísmo ou nos resíduos das regressões envolvendo os setores de atuação dos profissionais de Enfermagem. Além disso, a análise de Kruskal-Wallis revelou as diferenças significativas na mediana dos absenteísmos mensais entre os quatro setores avaliados (Emergência, CTI, Unidade de Internação Clínica e Unidade de Internação Cirúrgica). Especificamente, o teste de comparações múltiplas de Dunn mostrou que o absenteísmo-doença mensal mediano no setor de CTI foi significativamente diferente em comparação aos demais setores (Figura 1).

Na Tabela 2, estão apresentados os principais motivos de absenteísmo-doença entre os profissionais de Enfermagem, categorizados de acordo com os períodos analisados. A partir da análise dos dados, foram identificados seis principais motivos: doenças osteomusculares, psicossociais, infecções pulmonares (exceto COVID-19), condições inespecíficas, infecção pela COVID-19 e uma categoria abrangente denominada “Outros”. A categoria “Outros” reúne todas as condições que registraram uma frequência inferior a 5%, incluindo Gastrointestinal, Obstetrícia/Urologia/Ginecologia, Dermatologia, Oftalmologia, Cardiologia, Otorrinolaringologia, Vacinação, Oncologia, Câncer de Mama, Pré- e Pós-operatório, Doenças Crônicas, Neurologia, Pulmonar e Metabólica/Hematologia. Todos os motivos foram classificados conforme o Código Internacional de Doenças (CID-10) e organizados cronologicamente nos períodos pré-pandemia, durante a pandemia e pós-pandemia da COVID-19.

**Figura 1 -:** Taxa de absenteísmo-doença de acordo com os setores de atuação dos profissionais de Enfermagem afastados antes, durante e após a pandemia pela COVID-19 (n = 839). Porto Alegre, RS, Brasil, 2023-2024

**Tabela 2 -:** Testes de Qui-quadrado de Pearson e Análise de Variância (*One-Way*) aplicados aos motivos do absenteísmo-doença dos profissionais de Enfermagem de acordo com os períodos (n = 839). Porto Alegre, RS, Brasil, 2023-2024

Motivos de absenteísmo-doença	1º Período (Antes) n*= 477 ( *f* % ^†^ )	2º Período (Durante) n*= 665 ( *f* % ^†^ )	3º Período (Depois) n*= 699 ( *f* % ^†^ )	P ^‡^
COVID-19 (Sim)	34 ^§^ (7,1)	385 ^§^ (42,1)	275 ^§^ (39,3)	<0,001
(Não)	443 (92,9)	280 (57,9)	424 (60,7)	
Osteomuscular (Sim)	238 ^§^ (49,9)	292 ^§^ (43,9)	350 ^§^ (50,1)	<0,001
(Não)	239 (50,1)	373 (56,1)	349 (49,9)	
Psicossocial (Sim)	111 (23,3)	123 (18,5)	125 (17,9)	0,263
(Não)	366 (76,6)	542 (81,5)	574 (82,1)	
Pulmonar infeccioso (Sim)	129 ^§^ (27)	170 ^§^ (25,6)	249 ^§^ (35,6)	0,003
(Não)	348 (73)	495 (74,4)	450 (64,4)	
Inespecíficas (Sim)	268 ^§^ (43,8)	271 ^§^ (40,8)	321 ^§^ (45,9)	0,001
(Não)	209 (56,2)	394 (59,28)	378 (54,1)	
Outros (Sim)	222 ^§^ (46,5)	187 ^§^ (28,1)	264 ^§^ (37,8)	<0,001
(Não)	225 (53,5)	478 (71,9)	435 (62,5)	

*n = Total de Amostra; ^†^ *f*% = Porcentagem da Amostra; ^‡^P = p-valor; ^§^Representam subgrupos que diferem entre si a um valor de p<0,05

No que tange aos fatores de adoecimento dos profissionais de Enfermagem, na Tabela 3 são apresentadas as associações significativas dos motivos de absenteísmo-doença a partir do Modelo de Regressão de Poisson com variância robusta e conforme os períodos antes, durante e após a pandemia pela COVID-19.

**Tabela 3 -:** Modelo de regressão de Poisson com variância robusta para as variáveis associadas aos motivos de absenteísmo-doença dos profissionais de enfermagem nos períodos antes, durante e após a pandemia pela COVID-19 (n = 839). Porto Alegre, RS, Brasil, 2023-2024

**T** *	**Variáveis**	**COVID-19**		**Osteomuscular**		**Psicossocial**		**Infeccioso**		**Inespecífico**		**Outros**	
		RP ^†^ (95% IC ^‡^ )	P ^§^	RP ^†^ (95% IC ^‡^ )	P ^§^	RP ^†^ (95% IC ^‡^ )	P ^§^	RP ^†^ (95% IC ^‡^ )	P ^§^	RP ^†^ (95% IC ^‡^ )	P ^§^	RP ^†^ (95% IC ^‡^ )	P ^§^
**1º**	**Sexo**												
	Feminino									1			
	Masculino									0,234 (0,124-0,440)	0,001	0,342 (0,180-0,648)	0,001
	**Idade (anos)**												
	<= 41			1						1			
	42 a 46			1,242 (0,987-1,824)	0,06					0,604 (0,274-1,329)	0,21		
	47 a 52			1,332 (0,969-1,829)	0,077					1,477 (0,593-3,678)	0,402		
	>= 53			1,425 (1,036-1,961)	0,029					4,974 (1,502-16,47)	0,009		
	**Ocupação**												
	Enfermeiro(a)							1					
	Técnico(a) de Enfermagem							1,900 (1,187-3,040)	0,007				
	**Setor**												
	Emergência			1		1		1		1			
	Terapia Intensiva			0,677 (0,486-0,944)	0,021	0,415 (0,229-0,752)	0,004	0,547 (0,328-0,914)	0,021	1,974 (0,631-6,174)	0,242		
	Internação Clínica			1,036 (0,766-1,401)	0,82	0,837 (0,517-1,355)	0,47	1,197 (0,776-1,845)	0,416	3,482 (1,244-9,747)	0,017		
	Internação Cirúrgica			1,007 (0,738-1,373)	0,966	1,153 (0,726-1,830)	0,546	0,848 (0,523-1,376)	0,505	1,198 (0,533-2,691)	0,662		
**2º**	**Estado civil**												
	Com parceiro							1					
	Sem parceiro							0,643 (0,489-0,845)	0,002				
	**Ocupação**												
	Enfermeiro(a)			1									
	Técnico(a) de Enfermagem			1,341 (1,043-1,724)	0,022								
**3°**	**Sexo**												
	Feminino									1			
	Masculino									0,536 (0,339-0,848)	0,008		
	**Estado civil**												
	Com parceiro					1							
	Sem parceiro					0,691 (0,495-0,965)	0,03						
	**Ocupação**												
	Enfermeiro(a)			1		1							
	Técnico(a) de Enfermagem			1,456 (1,165-1,820)	0,001	1,756 (1,112-2,772)	0,016						
	**Setor**												
	Emergência	1		1									
	Terapia Intensiva	0,784 (0,608-1,011)	0,06	0,747 (0,593-0,941)	0,013								
	Internação Clínica	0,655 (0,496-0,865)	0,003	0,866 (0,691-1,086)	0,213								
	Internação Cirúrgica	0,701 (0528-0,930)	0,014	0,891 (0,709-1,820)	0,321								

*T = Período;^†^RP = Razão de Prevalência; ^‡^IC = Intervalo de Confiança; ^§^P = p-valor

## Discussão

Este estudo identificou e analisou os fatores relacionados ao absenteísmo-doença dos profissionais de Enfermagem brasileiros antes, durante e após a pandemia pela COVID-19. Evidenciou-se que fatores sociodemográficos, como a faixa etária e o número de dias perdidos, possuem associação com o absenteísmo destes profissionais. As taxas de absenteísmo aumentaram significativamente durante a pandemia e o setor da CTI apresentou um absenteísmo-doença mensal mediano significativamente diferente dos demais setores. Os motivos de adoecimento mais prevalentes foram doenças osteomusculares, COVID-19, outras infecções pulmonares e doenças inespecíficas.

Este estudo identificou aumento nas taxas de absenteísmo após a pandemia que superaram aquelas registradas no período pré-pandemia - embora tenham sido consideradas elevadas em todos os períodos. Alguns estudos internacionais já relataram um aumento rápido do absenteísmo e do adoecimento de profissionais durante a pandemia^(15,30-31)^. Contudo, ainda não existem estudos definitivos sobre o absenteísmo-doença após a pandemia. Pode-se supor que as elevadas taxas de absenteísmo pós-pandemia representem possíveis sequelas à saúde dos profissionais de Enfermagem, já adoecidos pelo trabalho durante a pandemia.

As maiores taxas foram concentradas nos períodos durante a pandemia no ano de 2020, com 9,3% em junho na Unidade de Internação Cirúrgica e 9,9% em julho na Unidade de Internação Clínica. Os valores encontrados demonstraram estar bem acima do limite esperado estabelecido pelo Conselho Fedral de Enfermagem (COFEN) de 6,7% de ausências não previstas, especialmente durante o período da pandemia, o que reforça o impacto significativo da pandemia na Enfermagem neste período^(32)^. Um outro estudo divergiu desses dados, pois identificou que a menor taxa mensal de absenteísmo foi no período anterior a pandemia, sendo 2,07% em dezembro de 2019, enquanto a maior taxa foi de 9,82% em julho de 2020^(33)^.

No período pré-pandemia, a faixa etária predominante foi de 53 anos ou mais, representando 123 (30,1%) casos, o maior percentual registrado nesse período. Em contrapartida, durante a pandemia, a faixa etária mais prevalente foi a de 41 anos ou menos, totalizando 202 (37,9%) casos, o que também se manteve no pós-pandemia, com 219 (41,1%). Ao alinhar-se a esses dados, alguns estudos realizados durante a pandemia demonstraram que a equipe de Enfermagem, em associação com o ambiente de trabalho, apresentou predominância na faixa etária de 36 a 40 anos, além de uma relação significativa com o absenteísmo^(10,34)^. Vale ressaltar que, durante a pandemia, havia orientações para que os indivíduos pertencentes aos grupos de risco se afastassem de suas atividades laborais; isso geralmente inclui as pessoas de idade mais avançada. Um estudo multicêntrico brasileiro reforça que a idade é um importante fator de risco entre os profissionais ativos durante a pandemia. Devido a esse risco de adoecimento por contaminação com o vírus, os profissionais pertencentes ao grupo de risco demonstraram preocupação durante suas atividades laborais, o que poderia levar a prejuízos psicológicos^(35)^.

No período anterior à pandemia, estudo realizado no Chile demonstrou que o cansaço físico aumentava em 1,05 vezes a probabilidade de absenteísmo laboral. Além disso, trabalhar por mais de um ano no mesmo serviço clínico aumentava o risco de faltar ao trabalho em 1,084 vezes^(36)^. Outro estudo, que analisou 2.761 afastamentos de profissionais de Enfermagem, revelou que 449 (16,26%) eram afastamentos relacionados a distúrbios osteomusculares. Neste estudo, o serviço com mais afastamentos foi da internação clínica, e o grupo com maior tempo de afastamento (> de 15 dias) foi composto por auxiliares e técnicos de Enfermagem (p=0,006), os trabalhadores com menor mediana de idade (p=0,021) e de maior escolaridade (p=0,035)^(37)^. Ademais, ser profissional da Unidade de Internação Clínica demonstrou uma probabilidade 3,48 vezes maior de afastamentos por doenças inespecíficas em comparação ao setor de Emergência, que apresentou a menor prevalência.

Durante a pandemia, a maior associação observada foi referente à ocupação, em que a probabilidade dos técnicos de Enfermagem foi 1,34 vez maior de absenteísmo por doenças osteomusculares, quando comparados aos enfermeiros. Em consonância com estes resultados, outros estudos apontaram que os técnicos de Enfermagem apresentaram maior índice de ausências em unidade de urgência e emergência^(34)^, bem como em enfermarias^(38)^. Um estudo realizado no Equador apontou que 85% dos auxiliares de Enfermagem sofrem de doenças osteomioarticulares, com maior incidência na região lombossacra e nos membros inferiores, durante sua jornada de trabalho. Além disso, observou-se que a elevada prevalência desses problemas de saúde está diretamente relacionada ao aumento do absenteísmo nas unidades de enfermaria. Foi identificado também que 39% dos participantes precisaram afastar-se temporariamente do trabalho por um período de um a três dias em razão de suas condições de saúde^(38)^.

Em contrapartida aos menores percentuais de absenteísmo-doença do setor Emergência neste estudo, outros autores apontaram o contexto em que esses profissionais operavam, caracterizado por sua natureza emergencial, variável e imprevisível, que exige uma carga de trabalho elevada e a gestão de casos complexos^(8)^. Isso requer o engajamento do profissional em atividades laborais que abrangem os aspectos físicos, mentais e psicossociais, dada a gama de fragilidades e vulnerabilidades associadas ao ambiente em que atuam. Destaca-se que quando as taxas de absenteísmo na Enfermagem são elevadas, fica mais complexo ajustar as escalas de trabalho para atender a todas as demandas exigidas pela jornada de trabalho.

Embora esta pesquisa não tenha encontrado associações significativas entre o absenteísmo-doença e os fatores psicossociais ou doenças psicossomáticas, alguns estudos já evidenciaram impactos relevantes na saúde mental de profissionais da saúde.

Um estudo canadense relacionou o impacto psicológico à junção de fatores, tanto da vida pessoal quanto da carga do ambiente laboral, especificamente no setor de cuidados intensivos. Nesse contexto, os enfermeiros vivenciaram um sofrimento psicológico significativo durante a pandemia da COVID-19^(11)^.

Em Atlanta, enfermeiros de UTI, um ambiente onde a Síndrome de *Burnout* já era comum entre a equipe multiprofissional antes da pandemia pela COVID-19, houve um aumento substancial na prevalência de esgotamento durante a pandemia^(39)^. Uma situação semelhante foi observada em profissionais de UTI no Brasil, que, devido à carga de trabalho excessiva, já apresentavam altos níveis de desgaste emocional e despersonalização no período anterior à pandemia^(40)^.

Um inquérito realizado com os profissionais de instituições de saúde mental na Holanda entre os anos de 2021 e 2022 revelou que a maioria dos entrevistados afirmou não sentir mais nem menos sintomas de ansiedade, depressão, estresse, tristeza e/ou raiva. No entanto, 35,7% (n = 182) dos entrevistados relataram “mais sintomas” de estresse, e 20,6% (n = 105) relataram o aumento nos sintomas de depressão em comparação ao período durante a pandemia. Esses dados indicam que tais sintomas foram significativamente mais intensos e prevalentes durante a pandemia em relação ao período pós-pandemia. Além disso, observou-se aumento no absenteísmo-doença no período pós-pandemia, acompanhado por maior frequência de ausências, resultados que se assemelham aos encontrados neste estudo^(41)^.

Na Jordânia, enfermeiras de unidades clínicas/cirúrgicas e de terapia intensiva apresentaram níveis de satisfação significativamente mais altos, com menores taxas de ausência e intenção de deixar o emprego durante a pandemia, quando comparados ao período pré-pandemia da COVID-19^(42)^. Um estudo multicêntrico, realizado com trabalhadores de Enfermagem de terapia intensiva em quatro hospitais de referência para o atendimento à COVID-19, revelou que a resiliência interfere positivamente nos domínios de desgaste emocional e a baixa realização profissional associados à Síndrome de *Burnout*. O estudo também destacou que o nível de exposição à COVID-19 influenciou significativamente a percepção do impacto da pandemia sobre a saúde mental dos profissionais^(43)^.

De acordo com outros estudos, as principais causas de absenteísmo durante a pandemia foram relacionadas à infecção pela COVID-19, problemas respiratórios, condições musculoesqueléticas, questões familiares, satisfação com a saúde, problemas de saúde mental^(7,44-45)^, com destaque para ansiedade, depressão e estresse^(10,18)^. Uma revisão integrativa revelou que 92% dos artigos analisados citaram doenças do sistema musculoesquelético, 64% relataram transtornos mentais e comportamentais/psicológicos, e 48% mencionaram doenças do sistema respiratório como as mais prevalentes^(4)^. Além disso, um estudo identificou que a inexperiência dos profissionais de Enfermagem de unidades dedicadas à COVID-19 representou um aumento de sobrecarga para os profissionais experientes, que tiveram que conduzir treinamentos e supervisões. Apesar disso, comprovou-se que esses profissionais, mesmo atuando em setores distintos, com diferentes níveis de exposição ao ambiente laboral, enfrentaram igualmente os prejuízos à saúde^(46)^.

É importante considerar algumas limitações ao interpretar os resultados deste estudo. Primeiramente, há a impossibilidade de generalizar os resultados para outras áreas e instituições, pois a pesquisa foi realizada em ambientes hospitalares de uma única instituição, que possui características próprias. Além disso, a presença de motivos de adoecimento classificados como inespecíficos pode tornar os dados mais suscetíveis a vieses.

Por fim, estes dados permitem que técnicos e enfermeiros reconheçam os fatores que influenciam o absenteísmo em sua rotina de trabalho, possibilitando a reflexão e instigando a implementação de ações que auxiliem na prevenção do adoecimento.

## Conclusão

Os fatores relacionados ao absenteísmo-doença dos profissionais de Enfermagem brasileiros antes, durante e após a pandemia pela COVID-19 foram estatisticamente associados à média geral de número de dias de ausência por profissional e à faixa etária de 41 anos ou menos. Ou seja, os profissionais que tiveram períodos de afastamento mais longos, assim como aqueles na fase adulto-jovem, apresentaram maiores chances de se ausentar do trabalho. Além disso, o número médio de dias perdidos foi significativamente maior durante o período pandêmico.

As taxas de absenteísmo durante e após a pandemia superaram as registradas no período anterior, embora tenham se mantido elevadas em todos os períodos analisados. Entre os principais motivos de absenteísmo-doença, destacaram-se a infecção por COVID-19 e as doenças osteomusculares.

A identificação dos aspectos relacionados ao absenteísmo-doença entre os profissionais de Enfermagem brasileiros traz contribuição significativa para o debate acadêmico sobre os impactos da pandemia na saúde ocupacional, especialmente em uma categoria profissional essencial como a Enfermagem. Os resultados deste estudo fornecem subsídios importantes para que os gestores desenvolvam políticas preventivas e corretivas mais eficazes, voltadas aos profissionais com múltiplos afastamentos ou períodos prolongados de ausência. No âmbito social, ressalta-se a importância de priorizar a saúde física e mental desses profissionais, considerando os riscos e os desafios inerentes à sua atividade laboral, particularmente em contextos de crise, como pandemias.

Dessa forma, este estudo reforça a necessidade de se implementar estratégias integradas que promovam o bem-estar desses profissionais, garantindo a sustentabilidade do sistema de saúde e a qualidade do atendimento à população.

# Translation: Factores relacionados con el ausentismo por enfermedad de los profesionales de enfermería brasileños antes, durante y después de la pandemia*

**DOI:** 10.1590/1518-8345.7696.4622

**Authors:** Fernanda Seidel Pinheiro, Miguel Lucas Silva da Paixão, Gabriel Fernandes Gonçalves, João Lucas Campos de Oliveira, Daiane Dal Pai, Juliana Petri Tavares

**Affiliations:** 1 Universidade Federal do Rio Grande do Sul, Escola de Enfermagem, Porto Alegre, RS, Brasil.

**Keywords:** Absentismo, Salud Laboral, Enfermería, COVID-19, Pandemias, Administración de Personal

## Abstract

identificar los factores relacionados con el ausentismo por enfermedad de los profesionales de enfermería brasileños antes, durante y después de la pandemia de COVID-19.

estudio transversal con profesionales de enfermería de internación clínica, quirúrgica, de unidad de cuidados intensivos y de emergencias para adultos, con ausencias entre 2019 y 2022. Se evaluaron las variables sociodemográficas, laborales y de ausentismo. Se realizó análisis estadístico descriptivo, cálculo de tasa de ausentismo y Regresión de Poisson con varianza robusta, considerando p≤0,05.

la muestra estuvo compuesta por 839 profesionales, con 7.375 ausencias. El ausentismo por enfermedad arrojó una media de 54,1±2,5 días perdidos (p<0,001) y profesionales de 41 años o menos (31,8%; p=0,003). Los sectores de cuidados intensivos (31,3%) e internación clínica (27,5%) fueron los que presentaron mayor ausencia. La tasa de ausentismo más alta (9,9%) se registró en julio de 2020. El riesgo de enfermedad se relacionó con el sexo masculino (p≤0,001) y cuidados intensivos (p=0,007) en el 1er periodo; solteros (p=0,002) y técnicos en enfermería (p=0,022) en el 2º periodo; y cuidados intensivos (p=0,003) y técnicos en enfermería (p≤0,001) en el 3er periodo.

cuando finalizó la pandemia, las tasas de ausentismo no volvieron a los parámetros prepandemia. La COVID-19 y las enfermedades musculoesqueléticas fueron las más prevalentes. Se pudo investigar los factores relacionados con el ausentismo.

## Destacados

**(1)** El número de días de ausencia y la edad ≤ 41 años se asocian con el ausentismo. **(2)** Los principales motivos del ausentismo por enfermedad fueron la COVID-19 y las enfermedades musculoesqueléticas. **(3)** La internación clínica presentó la tasa de ausentismo más alta de todos los sectores y períodos. **(4)** El ausentismo en el segundo y tercer período superó al del primero, pero fue alto en todos los períodos.

## Introducción

La discusión sobre enfermedad profesional es de larga data, pero es un problema constante, incluso cuando se trata de profesionales de enfermería. El ausentismo laboral de los profesionales de enfermería es un problema de gran relevancia para la salud pública y afecta directamente la calidad de la atención y la sostenibilidad de los servicios de salud. Se sabe que el ausentismo por enfermedad o procedimientos médicos incluye todas las ausencias por enfermedad o procedimientos médicos, con excepción de las enfermedades profesionales^(1)^. Cuando se presenta en los trabajadores de enfermería, el ausentismo desorganiza el servicio, provoca insatisfacción y sobrecarga en el equipo, lo que genera una reducción de la calidad de la atención que se le brinda a los pacientes^(2)^.

Los impactos psicosociales y psicosomáticos derivados de la naturaleza del trabajo de enfermería reducen la productividad y tienden a aumentar el trauma, el agotamiento emocional, el miedo al contagio, y sentimientos como tristeza, mal humor y ganas de renunciar a todo, que contribuyen al aumento del ausentismo de dichos profesionales en los servicios de salud^(3-6)^.

Entre los principales motivos del ausentismo de los profesionales de enfermería se encuentran las enfermedades del sistema respiratorio, enfermedades infecciosas y parasitarias, así como enfermedades del sistema musculoesquelético y del tejido conectivo, muchas veces debido a exposiciones ocupacionales^(7)^. Se destacan fundamentalmente las enfermedades mentales y del comportamiento, clínicas, musculoesqueléticas y del tejido conectivo, que generalmente presentan los porcentajes más elevados^(5,8)^.

Con la llegada de la pandemia de COVID-19, se maximizaron los problemas que afectaban cotidianamente a los profesionales de enfermería, como la sobrecarga de trabajo, las largas y agotadoras jornadas laborales, la mala calidad del sueño, las jornadas laborales dobles, los procesos de trabajo y los recursos materiales ineficaces, entre otros^(9-10)^. Ante la rápida propagación de la COVID-19, estudios nacionales e internacionales reportaron crecientes preocupaciones, entre ellas la anticipación del impacto de la enfermedad, el temor a la reducción o falta de Equipos de Protección Personal (EPP), además del temor por la propia seguridad y la de los seres queridos, que eran alarmantes^(11-12)^.

A nivel internacional, algunos estudios han identificado un mayor riesgo de morbilidad física grave y largos períodos de ausencia en los profesionales de enfermería que atendieron a pacientes con COVID-19^(13-15)^. También se observó una alta prevalencia de enfermedades psicológicas y varios profesionales desarrollaron síntomas de ansiedad, depresión, estrés postraumático y *Burnout* debido a las experiencias que tuvieron durante la pandemia^(10,16-18)^.

Algunos estudios realizados durante la pandemia de COVID-19 teorizaron que hubo un aumento de la gravedad clínica de los pacientes con enfermedades crónicas no transmisibles, dado que muchos no recibieron tratamiento regular debido al aislamiento^(19-20)^. Este fenómeno podría generar una nueva sobrecarga en el sistema sanitario, que tendría un impacto más grave sobre los profesionales que enfermaron debido a la pandemia, sobre todo porque no tuvieron tiempo suficiente para recuperarse.

Si bien la literatura presenta muchos de los desafíos que enfrentaron los profesionales de enfermería antes y durante la pandemia, aún hay una laguna en el conocimiento sobre los efectos postpandemia en la salud de estos trabajadores. Por ente, la realización de este estudio se justifica porque es necesario identificar el ausentismo laboral por enfermedad de los profesionales de enfermería brasileños en los períodos antes, durante y después de la pandemia de COVID-19. Además, es fundamental investigar los factores que contribuyeron al ausentismo de dichos profesionales en los diferentes sectores de atención hospitalaria, con el fin de discutir las acciones que se pueden implementar en estos contextos.

Por consiguiente, el objetivo del presente estudio fue identificar los factores relacionados con el ausentismo por enfermedad de los profesionales de enfermería brasileños antes, durante y después de la pandemia de COVID-19.

## Método

### Diseño del estudio

Se trata de un estudio transversal, retrospectivo, que se basa en la herramienta *Strengthening the Reporting of Observational Studies in Epidemiology* (STROBE)^(21)^.

### Escenario

El estudio se llevó a cabo en un hospital escuela cuaternario del sur de Brasil. La institución es pública, está vinculada a una universidad y atiende mediante el Sistema Único de Salud (SUS). Durante la pandemia de COVID-19, la institución se convirtió en referencia para la atención de alta complejidad a pacientes infectados. Precisamente, el estudio se basó en datos de ausencias de profesionales de Enfermería de los Servicios de Emergencias para Adultos, Unidades de Cuidados Intensivos (UCI) para Adultos, Unidades de Internación clínica y Unidades de Internación Quirúrgica.

### Período

En base al número de casos totales informado por el Consejo Nacional de Secretarios de Salud (CONASS), los períodos fueron designados como “Antes” de la pandemia, de marzo de 2019 a marzo de 2020, “Durante”, de abril de 2020 a abril de 2021, y “Después”, de mayo de 2021 a mayo de 2022^(22)^.

El período “Antes” se definió como el espacio temporal correspondiente a un año antes del inicio del aislamiento social en el estado de Rio Grande do Sul, de marzo de 2019 a marzo de 2020. Al final de este período, el estado tenía aproximadamente 1.000 casos confirmados de COVID-19. El periodo “Durante” se definió de abril de 2020 a abril de 2021, contemplando el aumento exponencial de casos y muertes en el estado. El período “Después” se estableció entre mayo de 2021 y mayo de 2022. En este contexto, ya se había determinado en el estado la distribución de vacunas a los profesionales de la salud^(23)^, y se observó un descenso en el número de muertes por COVID-19.

### Participantes

La población total disponible incluyó los 1.455 profesionales de Enfermería (enfermeros y técnicos en enfermería) contratados por los sectores investigados. De éstos, se obtuvo un total reducido a través de los datos de ausencia.

Los criterios de inclusión fueron: profesionales de enfermería (enfermeros y técnicos en enfermería), de ambos sexos, que tuvieran al menos una ausencia al trabajo por enfermedad o procedimiento médico entre marzo de 2019 y mayo de 2022. Se consideraron pérdidas, sin control, las ausencias ocasionadas por enfermedades no registradas.

Para los efectos de los análisis realizados, se incluyeron individuos con múltiples ausencias en cada período, así como individuos con ausencias en sólo uno de los períodos. Por ende, cada período presenta una población final diferente, representativa de todos los profesionales y sus ausencias dentro del tiempo descrito.

### Fuentes de datos y variables

La recolección de datos se realizó a través de una *Query* solicitada a la institución, que ya contaba con los mismos en una base de datos institucional. Los datos extraídos del Sector de Medicina Laboral (SML) y de la Coordinación de Gestión de Recursos Humanos (CGRH) fueron puestos a disposición de los autores de forma anonimizada. La institución proporcionó los datos brutos y la primera autora los compiló y organizó. Se tabularon las variables seleccionadas, incluyendo sexo, edad, etnia, cargo/función, sector de actividad, tiempo en la institución, infección por Covid-19, periodo y duración de la ausencia, motivo de la ausencia y CIE-10^(24)^ (Clasificación Estadística Internacional de Enfermedades y Problemas Relacionados con la Salud). A cada caso se le asignó un código numérico para identificar las historias clínicas de forma anónima.

Los motivos de ausentismo laboral por enfermedad, categorizados según la CIE-10, se subdividieron en seis categorías: COVID-19, Musculoesquelético/Traumatología, No específica, Psicosocial e Infecciones, y la categoría Otros, constituida por todas las categorías que aparecieron con una frecuencia inferior al 5%, que son: Gastrointestinal, Obstetricia/Urología/Ginecología, Dermatología, Oftalmología, Cardiología, Otorrinolaringología, Vacunación, Oncología, Cáncer de mama, Pre y Postoperatorio, Enfermedades crónicas, Neurología, Pulmonar, Metabólica/Hematología.

### Cálculo de la muestra

Considerando un plantel total disponible de hasta 1.455 profesionales, se estimó un tamaño de muestra de 732 profesionales de Enfermería (244 en cada grupo) para detectar diferencias significativas de Y entre los grupos A, B y C, las medias consideradas para cada grupo son 4,2, 5,6 y 4,2 u.m. (unidades de medida - días), respectivamente. Se adicionó un 10% por posibles pérdidas y se obtuvo que el número mínimo de individuos era 816. El cálculo consideró una potencia del 90%, un nivel de significación del 5% y una desviación estándar de 5 u.m. (días). Este cálculo se realizó mediante la herramienta *PSS Health*, versión en línea, con ayuda de un estadístico^(25)^. Se consideró que la muestra final debía estar compuesta por 839 profesionales y se alcanzó el número mínimo de individuos requerido.

### Variables cuantitativas y análisis estadístico

Los datos se organizaron y transportaron desde una hoja de cálculo *de Microsoft OfficeExcel* ^®^ al programa *Statistical Package for the Social Sciences* ^®^ (SPSS) versión 26.0 para *Windows* ^®^. El análisis descriptivo de los resultados se realizó mediante frecuencia absoluta y relativa (n;%), así como mediante medidas de tendencia central (media y mediana) y de variabilidad (desviación estándar y amplitud).

Para comprobar la distribución de las variables continuas se utilizó la prueba de Kolmogorov-Smirnov y la prueba de análisis de varianza de Friedman*.*

El análisis bivariado entre variables categóricas se realizó mediante la prueba Chi-cuadrado de Pearson. Para las variables continuas comparadas entre tres o más grupos, se realizó la prueba de Análisis de Varianza (*One-Way).*

La tasa de ausentismo se obtuvo a través del número de días perdidos, con una versión simplificada de la fórmula, que se utiliza internacionalmente^(26)^ y en las directrices brasileñas^(27)^. La fórmula se describe como: “Tasa de ausentismo = Total de días laborables ausentes x 100 / Total de días laborables en el período x Total de empleados en el sector”. Se calculó la tasa de ausentismo de cada mes analizado, así como la tasa de ausentismo media de cada uno de los tres periodos.

Para comparar las tasas de ausentismo entre sectores y periodos se aplicaron las pruebas de Autocorrelación (ACF), Autocorrelación Parcial (PACF) y el estadístico de Durbin-Watson, cuyos valores fueron cercanos o mayores a 1,5, e indican ausencia de autocorrelación significativa. Además, se utilizó la prueba de Kruskal-Wallis para evaluar las diferencias en las medianas de las tasas de ausentismo entre los sectores, seguida de la prueba de comparaciones múltiples de Dunn para identificar qué sectores presentaban diferencias significativas entre ellos.

El análisis de la fuerza de asociación de las variables sociodemográficas y ocupacionales en relación a los motivos de ausentismo por enfermedad (p<0,20) se realizó mediante el Modelo de Regresión de Poisson con Varianza Robusta, cuyo análisis multivariado fue representado por la Razón de Prevalencia (RP) e Intervalos de Confianza (IC 95%). Para los criterios de decisión estadística, todos los análisis adoptaron un nivel de significación del 5%.

### Sesgo

Uno de los posibles sesgos de este manuscrito es la posibilidad de que haya habido errores de tipeo y tabulación de los datos durante los análisis. Para evitar esto, se realizó un doble control con ayuda de dos investigadores diferentes involucrados en el estudio. Además, la presencia de los motivos de enfermedad clasificados como inespecíficos puede hacer que los datos sean más susceptibles a sesgos.

### Aspectos éticos

El estudio fue presentado y aprobado por el Comité de Ética en Investigación (CEP/UFRGS), bajo el CAAE: 69221923.0.0000.5327. Se firmó el término de compromiso de uso de datos y se aceptó que no era necesario el Formulario de Consentimiento Libre e Informado (FCLI), debido a la anonimización de los datos recolectados. Por lo tanto, se respetaron los principios éticos establecidos en la Resolución N° 466/2012 del Consejo Nacional de Salud^(28)^ y la Ley General de Protección de Datos (LGPD), Ley N° 13.709, de agosto de 2018^(29)^.

## Resultados

Fueron 839 los profesionales que presentaron ausentismo por enfermedad en el período analizado. De los mismos, n=477 individuos estuvieron ausentes en el período “antes” de la pandemia, n=665 en el período “durante” y n=699 en el período “después”.

A partir de la muestra, se identificaron 7.375 ausencias por ausentismo laboral por enfermedad entre el período de marzo de 2019 y mayo de 2022. De dichas ausencias, 1.855 (25,2%) ocurrieron en el período antes de la pandemia; 2.551 (34,6%) durante la pandemia; y 2.969 (40,3%) después de la pandemia.

Los resultados generales identificaron que predominaron los profesionales del sexo femenino (79,6%; n=668), de etnia blanca (84,5%; n=709) y con edad igual o inferior a 41 años (31,8%; n=267). En lo que respecta al estado civil, la mayoría de los profesionales no tenía pareja (72,6%; n=609). En cuanto a la ocupación, los técnicos en enfermería presentaron mayor porcentaje de ausencias (76,2%; n=639) que los enfermeros (27,4%; n=200).

La distribución de los profesionales ausentes por sector de actividad es la siguiente: Emergencias (17,4%; n=146), UCI (31,3%; n=263), Unidad de Internación clínica (27,5%; n=231) y Unidad de Internación Quirúrgica (23,7%; n=199). La media de días perdidos por profesional de enfermería fue de 54,1 (DE=2,5).

Los factores estadísticamente asociados al ausentismo por enfermedad de los profesionales de enfermería fueron el número de días perdidos por profesional (p<0,001) y la franja etaria (p=0,003). Las demás variables no mostraron diferencia estadísticamente significativa en los periodos antes, durante y después de la pandemia de COVID-19, como se muestra en la Tabla 1.

**Tabla 1 -:** Pruebas de Kolmogorov-Smirnov y Análisis de Varianza de Friedman aplicadas a la caracterización de los profesionales de enfermería ausentes, antes, durante y después de la pandemia de COVID-19 (n = 839). Porto Alegre, RS, Brasil, 2023-2024

Variables	Total n=839 ( *f* )	1er Periodo (Antes) n=477 ( *f* )	2do Periodo (Durante) n=665 ( *f* )	3er Periodo (Después) n=699 ( *f* )	valor p
N° de días perdidos por profesional*	54,1 (σ=2,5)	15,05 ^†^ (σ=1,7)	20,2 ^†^ (σ=0,9)	18,8 ^†^ (σ=1,2)	<0,001
Sexo					0,655
Femenino	668 (79,6)	387 (81,1)	525 (78,9)	560 (80,1)	
Masculino	171 (20,4)	90 ^†^ (18,9)	140 (21,1)	139 (19,9)	
Franja etaria					0,003
<41 años	267 (31,8)	112 ^†^ (21,0)	202 ^†^ (37,9)	219 ^†^ (41,1)	
de 42 a 46 años	221 (26,3)	129 ^†^ (26,3)	174 ^†^ (35,5)	187 ^†^ (38,2)	
de 47 a 52 años	180 (21,5)	113 ^†^ (27,6)	147 ^†^ (35,6)	149 ^†^ (36,4)	
≥53 años	171 (20,4)	123 ^†^ (30,1)	142 ^†^ (34,7)	144 ^†^ (35,2)	
Etnia					0,977
Blanca	709 (84,5)	409 (26,0)	570 (36,6)	592 (37,7)	
Negra	100 (11,9)	54 (25,6)	74 (35,1)	83 (39,3)	
Mulata	30 (3,6)	14 (23,7)	21 (35,6)	24 (40,7)	
Estado civil					0,416
Con pareja	230 (27,4)	151 (28,0)	192 (35,6)	197 (36,5)	
Sin pareja	609 (72,6)	326 (25,1)	473 (36,4)	502 (38,6)	
Ocupación					0,969
Enfermero(a)	200 (23,8)	111 (26,2)	151 (35,6)	162 (38,2)	
Técnico(a) en enfermería	639 (76,2)	366 (76,7)	514 (36,3)	537 (37,9)	
Sector de actividad					0,234
Emergencia	146 (17,4)	85 (26,3)	113 (35,0)	125 (38,7)	
UCI	263 (31,3)	116 (21,6)	209 (38,9)	212 (39,5)	
Internación Clínica	231 (27,5)	148 (28,2)	184 (35,0)	193 (36,8)	
Internación Quirúrgica	199 (23,7)	128 (28,1)	159 (34,9)	169 (37,1)	

*Media y desviación estándar; ^†^Representa subgrupos que difieren entre sí en un valor p<0,05

Antes de la pandemia, las tasas de ausentismo más altas en los sectores fueron 4,1% en el Departamento de Emergencias en marzo de 2020, 7,5% en la UCI en enero de 2020, 6,2% en la Unidad de Internación clínica en octubre de 2019 y 5% en la Unidad de Internación Quirúrgica en marzo de 2020. Durante la pandemia, el Departamento de Emergencias registró un aumento y llegó al 8,2% en abril de 2021. La Unidad de Internación Clínica alcanzó el 9,9% en julio de 2020, sin embargo, la UCI mostró una reducción, y su tasa más alta fue del 4,3% en junio de 2020. Por el contrario, la Unidad de Internación Quirúrgica alcanzó el 9,3% en junio de 2020. En el período pospandemia, el Departamento de Emergencias registró el 8,2% en agosto de 2021, la UCI presentó el 5% en abril de 2022, la Internación Clínica se mantuvo alta en 7,8%, en mayo de 2022, y la tasa de ausentismo más alta en la Unidad de Internación Quirúrgica fue del 6,6%, en junio de 2021.

Los Gráficos de Autocorrelación (ACF) y Autocorrelación Parcial (PACF), junto con el estadístico de Durbin-Watson, indicaron que no existe evidencia de autocorrelación en las series de ausentismo ni en los residuos de las regresiones que involucran los sectores en los que trabajan los profesionales de Enfermería. Además, el análisis de Kruskal-Wallis reveló las diferencias significativas en la mediana de los ausentismos mensuales entre los cuatro sectores evaluados (Emergencias, UCI, Unidad de Internación Clínica y Unidad de Internación Quirúrgica). En definitiva, la prueba de comparaciones múltiples de Dunn mostró que el ausentismo laboral mensual mediano por enfermedad en la UCI era significativamente diferente en comparación con los otros sectores (Figura 1).

En la Tabla 2 se presentan los principales motivos de ausentismo por enfermedad de los profesionales de enfermería, categorizados según los períodos analizados. A partir del análisis de los datos, se identificaron seis motivos principales: enfermedades musculoesqueléticas, enfermedades psicosociales, infecciones pulmonares (excepto COVID-19), afecciones no específicas, infección por COVID-19 y una categoría amplia denominada “Otros”. La categoría “Otros” abarca todas las afecciones que registraron una frecuencia menor al 5%, e incluyen Gastrointestinal, Obstetricia/Urología/Ginecología, Dermatología, Oftalmología, Cardiología, Otorrinolaringología, Vacunación, Oncología, Cáncer de Mama, Pre y Postoperatorio, Enfermedades Crónicas, Neurología, Pulmonar y Metabólica/Hematología. Todos los motivos se clasificaron según la Clasificación Internacional de Enfermedades (CIE-10) y se organizaron cronológicamente en los períodos prepandémico, pandémico y pospandémico de COVID-19.

**Figura 1 -:** Tasa de ausentismo por enfermedad por sectores de actividad de los profesionales de Enfermería ausentes antes, durante y después de la pandemia de COVID-19 (n = 839). Porto Alegre, RS, Brasil, 2023-2024

**Tabla 2 -:** Pruebas Chi-cuadrado de Pearson y Análisis de Varianza (*One-Way*) aplicadas a los motivos de ausentismo por enfermedad de los profesionales de Enfermería según los periodos (n = 839). Porto Alegre, RS, Brasil, 2023-2024

Motivos de ausentismo por enfermedad	1er período (Antes) n*=477 ( *f* % ^†^ )	2do Periodo(Durante)n*=665 ( *f* % ^†^ )	3er período(Después)n*=699 ( *f* % ^†^ )	p ^‡^
COVID-19 (Sí)	34 ^§^ (7,1)	385 ^§^ (42,1)	275 ^§^ (39,3)	<0,001
(No)	443 (92,9)	280 (57,9)	424 (60,7)	
Músculoesquelético (Sí)	238 ^§^ (49,9)	292 ^§^ (43,9)	350 ^§^ (50,1)	<0,001
(No)	239 (50,1)	373 (56,1)	349 (49,9)	
Psicosocial (Sí)	111 (23,3)	123 (18,5)	125 (17,9)	0,263
(No)	366 (76,6)	542 (81,5)	574 (82,1)	
Pulmonar infeccioso (Sí)	129 ^§^ (27)	170 ^§^ (25,6)	249 ^§^ (35,6)	0,003
(No)	348 (73)	495 (74,4)	450 (64,4)	
No específico (Sí)	268 ^§^ (43,8)	271 ^§^ (40,8)	321 ^§^ (45,9)	0,001
(No)	209 (56,2)	394 (59,28)	378 (54,1)	
Otros (Sí)	222 ^§^ (46,5)	187 ^§^ (28,1)	264 ^§^ (37,8)	<0,001
(No)	225 (53,5)	478 (71,9)	435 (62,5)	

*n = Muestra total; ^†^ *f* % = Porcentaje de la muestra; ^‡^p = Valor p; ^§^Representa subgrupos que difieren entre sí en un valor de p<0,05

En lo que respecta a los factores que conllevan a enfermedad en los profesionales de enfermería, en la Tabla 3 se presentan las asociaciones significativas de los motivos de ausentismo por enfermedad en base al Modelo de Regresión de Poisson con varianza robusta y según los periodos antes, durante y después de la pandemia de COVID-19.

**Tabla 3 -:** Modelo de regresión de Poisson con varianza robusta para las variables asociadas a los motivos de ausentismo por enfermedad de los profesionales de enfermería en los periodos antes, durante y después de la pandemia de COVID-19 (n = 839). Porto Alegre, RS, Brasil, 2023-2024

**T** *	**Variables**	**COVID-19**		**Músculoesquelético**		**Psicosocial**		**Infeccioso**		**No específico**		**Otros**	
		RP ^†^ (IC ^‡^ 95%)	p ^§^	RP ^†^ (IC ^‡^ 95%)	p ^§^	RP ^†^ (IC ^‡^ 95%)	p ^§^	RP ^†^ (IC ^‡^ 95%)	p ^§^	RP ^†^ (IC ^‡^ 95%)	p ^§^	RP ^†^ (IC ^‡^ 95%)	p ^§^
**1º**	**Sexo**												
	Femenino									1			
	Masculino									0,234(0,124-0,440)	0,001	0,342(0,180-0,648)	0,001
	**Edad (años)**												
	<41			1						1			
	42 a 46			1,242(0,987-1,824)	0,06					0,604(0,274-1,329)	0,21		
	47 a 52			1,332(0,969-1,829)	0,077					1,477(0,593-3,678)	0,402		
	≥53			1,425(1,036-1,961)	0,029					4,974(1,502-16,47)	0,009		
	**Ocupación**												
	Enfermero(a)							1					
	Técnico(a) en enfermería							1,900(1,187-3,040)	0,007				
	**Sector**												
	Emergencia			1		1		1		1			
	Cuidados Intensivos			0,677(0,486-0,944)	0,021	0,415(0,229-0,752)	0,004	0,547(0,328-0,914)	0,021	1,974(0,631-6,174)	0,242		
	Internación Clínica			1,036(0,766-1,401)	0,82	0,837(0,517-1,355)	0,47	1,197(0,776-1,845)	0,416	3,482(1,244-9,747)	0,017		
	Internación Quirúrgica			1,007(0,738-1,373)	0,966	1,153(0,726-1,830)	0,546	0,848(0,523-1,376)	0,505	1,198(0,533-2,691)	0,662		
**2º**	**Estado civil**												
	Con pareja							1					
	Sin pareja							0,643(0,489-0,845)	0,002				
	**Ocupación**												
	Enfermero(a)			1									
	Técnico(a) en enfermería			1,341(1,043-1,724)	0,022								
**3º**	**Sexo**												
	Femenino									1			
	Masculino									0,536(0,339-0,848)	0,008		
	**Estado civil**												
	Con pareja					1							
	Sin pareja					0,691(0,495-0,965)	0,03						
	**Ocupación**												
	Enfermero(a)			1		1							
	Técnico(a) en enfermería			1,456(1,165-1,820)	0,001	1,756(1,112-2,772)	0,016						
	**Sector**												
	Emergencia	1		1									
	Cuidados Intensivos	0,784(0,608-1,011)	0,06	0,747(0,593-0,941)	0,013								
	Internación Clínica	0,655(0,496-0,865)	0,003	0,866(0,691-1,086)	0,213								
	Internación Quirúrgica	0,701(0528-0,930)	0,014	0,891(0,709-1,820)	0,321								

*T = Periodo; ^†^RP = Razón de Prevalencia; ^‡^IC = Intervalo de Confianza; ^§^p = Valor p

## Discusión

Este estudio identificó y analizó los factores relacionados con el ausentismo por enfermedad de los profesionales de enfermería brasileños antes, durante y después de la pandemia de COVID-19. Se observó que factores sociodemográficos como la franja etaria y el número de días perdidos tienen asociación con el ausentismo laboral de estos profesionales. Las tasas de ausentismo aumentaron significativamente durante la pandemia y el sector de la UCI tuvo un ausentismo laboral por enfermedad mensual mediano significativamente diferente al de los otros sectores. Los motivos de enfermedad más prevalentes fueron enfermedades musculoesqueléticas, COVID-19, otras infecciones pulmonares y enfermedades no específicas.

Este estudio identificó aumentos en las tasas de ausentismo después de la pandemia que superaron las registradas en el período prepandémico, aunque se consideraron altas en todos los períodos. Algunos estudios internacionales ya informaron que hubo un rápido aumento del ausentismo y la enfermedad de los profesionales durante la pandemia^(15,30-31)^. Sin embargo, todavía no existen estudios definitivos sobre el ausentismo por enfermedad después de la pandemia. Se puede asumir que las altas tasas de ausentismo postpandemia representan posibles consecuencias para la salud de los profesionales de Enfermería, que ya habían enfermado debido al trabajo durante la pandemia.

Las tasas más altas se concentraron en los periodos durante la pandemia en 2020, 9,3% en junio en la Unidad de Internación Quirúrgica y 9,9% en julio en la Unidad de Internación Clínica. Los valores encontrados estuvieron muy por encima del límite esperado establecido por el Consejo Federal de Enfermería (COFEN) del 6,7% de ausencias imprevistas, especialmente durante el período de la pandemia, lo que destaca el impacto significativo que tuvo la pandemia en los profesionales de enfermería durante dicho período^(32)^. Otro estudio arrojó diferentes datos, dado que identificó que la tasa de ausentismo mensual más baja se presentó en el período anterior a la pandemia, 2,07% en diciembre de 2019, mientras que la tasa más alta fue del 9,82% en julio de 2020^(33)^.

En el período prepandemia, la franja etaria predominante fue de 53 años o más, y representó 123 (30,1%) casos, el mayor porcentaje registrado en este período. En cambio, durante la pandemia, la franja etaria más prevalente fue 41 años o menos, con 202 (37,9%) casos, que se mantuvo también después de la pandemia, con 219 (41,1%). De forma similar, algunos estudios realizados durante la pandemia demostraron que en el equipo de Enfermería, en asociación con el ambiente laboral, predominó la franja etaria de 36 a 40 años, y que hubo una relación significativa con el ausentismo laboral^(10,34)^. Cabe señalar que, durante la pandemia, se establecieron pautas para que las personas pertenecientes a grupos de riesgo se mantuvieran alejadas de sus actividades laborales, esto generalmente incluye a las personas mayores. Un estudio multicéntrico brasileño destaca que la edad es un factor de riesgo importante para los profesionales activos durante la pandemia. Debido al riesgo de enfermarse por contagio con el virus, los profesionales que pertenecían al grupo de riesgo mostraron preocupación durante sus actividades laborales, lo que podría provocar daños psicológicos^(35)^.

En el período previo a la pandemia, un estudio realizado en Chile reveló que el cansancio físico aumentaba 1,05 veces la probabilidad de ausentismo laboral. Además, trabajar durante más de un año en el mismo servicio clínico aumentaba el riesgo de faltar al trabajo 1,084 veces^(36)^. Otro estudio, que analizó 2.761 ausencias de profesionales de enfermería, reveló que 449 (16,26%) eran ausencias relacionadas con trastornos musculoesqueléticos. Durante ese estudio, el servicio con mayor número de ausencias fue el de internación clínica, y el grupo con mayor tiempo de ausencia (>15 días) estuvo compuesto por auxiliares y técnicos en enfermería (p=0,006), trabajadores con menor mediana de edad (p=0,021) y mayor nivel educativo (p=0,035)^(37)^. Por otro lado, ser profesional en la Unidad de Internación Clínica arrojó una probabilidad 3,48 veces mayor de ausencias por enfermedades inespecíficas que el sector de Emergencias, que presentó la menor prevalencia.

Durante la pandemia, la mayor asociación observada se relacionó con la ocupación, en la que los técnicos en enfermería tuvieron 1,34 veces más probabilidades de ausentarse por enfermedades musculoesqueléticas que los enfermeros. De forma similar, otros estudios mostraron que los técnicos en enfermería tenían una mayor tasa de ausencias en las unidades de urgencia y emergencia^(34)^ así como en las salas de internación general^(38)^. Un estudio realizado en Ecuador demostró que el 85% de los auxiliares de enfermería padecen enfermedades osteomioarticulares, con mayor incidencia en la región lumbosacra y miembros inferiores, durante su jornada laboral. Además, se observó que la alta prevalencia de estos problemas de salud está directamente relacionada con el aumento del ausentismo en las unidades de enfermería. También se identificó que el 39% de los participantes necesitaron ausentarse temporalmente del trabajo por un período de uno a tres días debido a sus condiciones de salud^(38)^.

En el presente estudio los porcentajes de ausentismo por enfermedad en el sector de Emergencias fueron menores, mientras que, por el contrario, otros autores señalaron que el contexto en el que operan dichos profesionales, que es de carácter urgente, variable e impredecible, les exige una elevada carga de trabajo y el manejo de casos complejos^(8)^. Ello requiere que el profesional se involucre en actividades laborales que abarcan aspectos físicos, mentales y psicosociales, debido a la gama de debilidades y vulnerabilidades asociadas al entorno en el que trabaja. Cabe señalar que cuando los índices de ausentismo de los profesionales de enfermería son elevados es más complejo ajustar los horarios de trabajo para satisfacer todas las demandas que implica la jornada laboral.

Aunque esta investigación no encontró asociaciones significativas entre el ausentismo laboral por enfermedad y factores psicosociales o enfermedades psicosomáticas, algunos estudios ya han demostrado los impactos relevantes en la salud mental de los profesionales de la salud.

Un estudio canadiense relacionó el impacto psicológico con una combinación de factores, tanto de la vida personal como del entorno laboral, específicamente en el sector de cuidados intensivos. En este contexto, los enfermeros experimentaron un sufrimiento psicológico significativa durante la pandemia de COVID-19^(11)^.

En Atlanta, los enfermeros de la UCI, un entorno donde el Síndrome de *Burnout* ya era común en el equipo multidisciplinario antes de la pandemia de COVID-19, presentaron un aumento sustancial de la prevalencia de agotamiento durante la pandemia^(39)^. Una situación similar se observó en los profesionales de UCI en Brasil, quienes, debido a la excesiva carga de trabajo, ya presentaban altos niveles de agotamiento emocional y despersonalización en el período previo a la pandemia^(40)^.

Una encuesta realizada a profesionales de instituciones de salud mental en los Países Bajos entre 2021 y 2022 reveló que la mayoría de los encuestados afirmaron no sentir más ni menos síntomas de ansiedad, depresión, estrés, tristeza y/o ira. Sin embargo, el 35,7% (n=182) de los encuestados informó “más síntomas” de estrés y el 20,6% (n=105) informó un aumento de los síntomas de depresión con respecto al período de la pandemia. Estos datos indican que dichos síntomas fueron significativamente más intensos y prevalentes durante la pandemia que durante el período pospandémico. Además, se observó un aumento del ausentismo por enfermedad en el período postpandemia, acompañado de una mayor frecuencia de ausencias, resultados que son similares a los encontrados en este estudio^(41)^.

En Jordania, las enfermeras de las unidades clínicas/quirúrgicas y de cuidados intensivos informaron niveles significativamente más altos de satisfacción, con tasas más bajas de ausencia e intención de dejar sus trabajos durante la pandemia, en comparación con el período anterior a la pandemia de COVID-19^(42)^. Un estudio multicéntrico, realizado con trabajadores de enfermería de cuidados intensivos en cuatro hospitales de referencia para la atención de COVID-19, reveló que la resiliencia interfiere positivamente en los dominios de agotamiento emocional y bajo logro profesional asociados al Síndrome *de Burnout*. El estudio también destacó que el nivel de exposición a la COVID-19 influyó significativamente en la percepción del impacto de la pandemia en la salud mental de los profesionales^(43)^.

Según otros estudios, los principales motivos de ausentismo laboral durante la pandemia estuvieron relacionadas con la infección por COVID-19, problemas respiratorios, afecciones musculoesqueléticas, problemas familiares, satisfacción con la salud, problemas de salud mental^(7,44-45)^, principalmente ansiedad, depresión y estrés^(10,18)^. Una revisión integradora reveló que el 92% de los artículos analizados citaban enfermedades del sistema musculoesquelético, el 64% informaban trastornos mentales y conductuales/psicológicos y el 48% mencionaban que las enfermedades más prevalentes eran las del sistema respiratorio^(4)^. Además, un estudio identificó que la inexperiencia de los profesionales de enfermería en unidades dedicadas a la COVID-19 representó una mayor sobrecarga para los profesionales experimentados, quienes tuvieron que realizar capacitaciones y supervisión. A pesar de ello, se ha comprobado que estos profesionales, incluso trabajando en sectores diferentes, con distintos niveles de exposición al ambiente laboral, se enfrentaron de la misma forma a los problemas de salud^(46)^.

Es importante considerar algunas limitaciones al interpretar los resultados de este estudio. En primer lugar, es imposible generalizar los resultados en otras áreas e instituciones, ya que la investigación se llevó a cabo en entornos hospitalarios de una única institución, que tiene sus propias características. Por otro lado, la presencia de motivos de enfermedad clasificados como inespecíficos puede hacer que los datos sean más susceptibles a sesgos.

Por último, estos datos les permiten a los técnicos y enfermeros reconocer los factores que influyen en el ausentismo en su rutina de trabajo, lo que posibilita la reflexión y promueve la implementación de acciones que contribuyan a prevenir enfermedades.

## Conclusión

Los factores relacionados con el ausentismo por enfermedad de los profesionales de enfermería brasileños antes, durante y después de la pandemia de COVID-19 se asociaron estadísticamente con la media general de días de ausencia por profesional y con la franja etaria de 41 años o menos. En otras palabras, los profesionales que tenían períodos de ausencia más largos, así como aquellos en la fase de adultos jóvenes, tenían más probabilidades de ausentarse del trabajo. Además, el número promedio de días perdidos fue significativamente mayor durante el período de la pandemia.

Las tasas de ausentismo durante y después de la pandemia superaron las registradas en el período anterior, aunque se mantuvieron elevadas en todos los períodos analizados. Entre los principales motivos de ausentismo por enfermedad se destacaron la infección por COVID-19 y las enfermedades musculoesqueléticas.

Identificar los aspectos relacionados con el ausentismo por enfermedad de los profesionales de enfermería brasileños contribuye significativamente al debate académico sobre los impactos de la pandemia en la salud ocupacional, especialmente en una categoría profesional esencial como la enfermería. Los resultados de este estudio brindan información importante para que los gestores desarrollen políticas preventivas y correctivas más efectivas, dirigidas a profesionales con múltiples ausencias o periodos prolongados de ausencia. En el ámbito social, se destaca la importancia de priorizar la salud física y mental de estos profesionales, considerando los riesgos y desafíos inherentes a su actividad laboral, particularmente en contextos de crisis, como las pandemias.

Por lo tanto, este estudio destaca que es necesario implementar estrategias integradas que promuevan el bienestar de dichos profesionales, y garanticen la sostenibilidad del sistema de salud y la calidad de la atención que se le brinda a la población.
